# Healthcare-associated infection and its determinants in Ethiopia: A systematic review and meta-analysis

**DOI:** 10.1371/journal.pone.0241073

**Published:** 2020-10-23

**Authors:** Abebaw Yeshambel Alemu, Aklilu Endalamaw, Demeke Mesfin Belay, Demewoz Kefale Mekonen, Biniam Minuye Birhan, Wubet Alebachew Bayih

**Affiliations:** 1 Department of Paediatrics and Neonatal Health Nursing, College of Health Sciences, Debre Tabor University, Debre Tabor, Ethiopia; 2 Department of Paediatrics and Child Health Nursing, School of Health Sciences, College of Medicine and Health Sciences, Bahir Dar University, Bahir Dar, Ethiopia; University of Ghana College of Health Sciences, GHANA

## Abstract

**Background:**

Healthcare-associated infection is a global threat in healthcare which increases the emergence of multiple drug-resistant microbial infections. Hence, continuous surveillance data is required before or after patient discharge from health institutions though such data is scarce in developing countries. Similarly, ongoing infection surveillance data are not available in Ethiopia. However, various primary studies conducted in the country showed different magnitude and determinants of healthcare-associated infection from 1983 to 2017. Therefore, this systematic review and meta-analysis aimed to estimate the national pooled prevalence and determinants of healthcare-associated infection in Ethiopia.

**Methods:**

We searched PubMed, Science Direct, Google Scholar, and grey literature deposited at Addis Ababa University online repository. The quality of studies was checked using Joanna Brigg’s Institute quality assessment scale. Then, the funnel plot and Egger’s regression test were used to assess publication bias. The pooled prevalence of healthcare-associated infection was estimated using a weighted-inverse random-effects model meta-analysis. Finally, the subgroup analysis was done to resolve the cause of statistical heterogeneity.

**Results:**

A total of 19 studies that satisfy the quality assessment criteria were considered in the final meta-analysis. The pooled prevalence of healthcare-associated infection in Ethiopia as estimated from 18 studies was 16.96% (95% CI: 14.10%-19.82%). In the subgroup analysis, the highest prevalence of healthcare-associated infection was in the intensive care unit 25.8% (95% CI: 3.55%-40.06%) followed by pediatrics ward 24.16% (95% CI: 12.76%-35.57%), surgical ward 23.78% (95% CI: 18.87%-29.69%) and obstetrics ward 22.25% (95% CI: 19.71%-24.80%). The pooled effect of two or more studies in this meta-analysis also showed that patients who had surgical procedures (AOR = 3.37; 95% CI: 1.85–4.89) and underlying non-communicable disease (AOR = 2.81; 95% CI: 1.39–4.22) were at increased risk of healthcare-associated infection.

**Conclusions:**

The nationwide prevalence of healthcare-associated infection has remained a problem of public health importance in Ethiopia. The highest prevalence was observed in intensive care units followed by the pediatric ward, surgical ward and obstetrics ward. Thus, policymakers and program officers should give due emphasis on healthcare-associated infection preventive strategies at all levels. Essentially, the existing infection prevention and control practices in Ethiopia should be strengthened with special emphasis for patients admitted to intensive care units. Moreover, patients who had surgical procedures and underlying non-communicable diseases should be given more due attention.

## Introduction

According to the Communicable Diseases Control (CDC), healthcare-associated infection (HCAI) is defined as the acquisition of infectious agent(s) or its toxin(s) which occurs after 48 hours of hospital admission, or up to 3 days after discharge, or up to 30 days after the operation when someone was admitted for reasons other than infection [1, 2].

Globally, according to the World Health Organization (WHO) 2019 HCAI fact sheet report, a hundred million patients were affected each year [3]. The point prevalence of HCAI ranged from 3.5%-12% and 5.7%-19.1% in developed and Low-and Middle-Income Countries (LMICs), respectively [3, 4]. Though data is scarce, the burden of HCAI was found to be high in Sub-Saharan Africa (SSA) countries [5]. Specifically, the prevalence of HCAI was noted in Botswana (13.4%) [6], South Africa (8%) [7], and Ethiopia (13% to 22%) [8–10].

Healthcare-associated infection increases the occurrence of antimicrobial resistance [11], long-term disability [4], and mortality among individual patients [12]. The additional financial burden to the healthcare system, patients, and families due to HCAI is also significant [4]. Hence, the “*clean care the safer care*” program has been launched in 2004 with the WHO patient safety directive, which was aimed to reduce HCAI through improving hand hygiene practice at the center of achieving its aim [13]. The aforementioned infection prevention program and the WHO initiative about infection prevention and control policy recommendations have been implemented in developing countries, including Ethiopia. Despite these efforts, studies conducted at different settings of the globe revealed that admission to the surgical ward and hospital type [8], chest tube placement, prolonged hospital stays, patient on mechanical ventilation, previous hospitalization [9], pediatric patients, malnutrition, and length of staying in hospital >5days [10] were contributing factors of HCAI.

Various studies were conducted to determine the prevalence of HCAI in Ethiopia, but it showed great variation across geographical setting and variant periods. Based on this fact, there was a need for nationally representative data on HCAI in the country. Moreover, the pooled effect sizes of the determinants of HCAI weren’t explored nationwide. Consequently, this systematic review and meta-analysis was aimed to address the following research questions: (1) what is the national pooled prevalence of HCAI in Ethiopia; and (2) what are the determinants of HCAI in the country?

## Materials and methods

### Reporting

The study results were reported based on the Preferred Reporting Items for Systematic Review and Meta-analysis statement (PRISMA) guideline [14] (S1 File). The protocol was registered on the PROSPERO database with a registration number (CRD42020166761), and available on https://www.crd.york.ac.uk/prospero/display_record.php?ID=CRD42020166761.

### Inclusion and exclusion criteria

We included cross-sectional, case-control and cohort studies, but case-control studies weren’t used to estimate the pooled prevalence of HCAI. These studies were included when the prevalence, incidence, and/or at least one determinant was reported. All studies published in the English language were considered. There was no restriction of the study period, age group, and study setting. All citations without abstract and/or full-text, anonymous reports, editorials, and qualitative studies were excluded.

### Search strategy and information source

PubMed, Science Direct, Google Scholar, and grey literature deposited at Addis Ababa University online repository were searched. The core search terms and phrases were “prevalence”, “incidence”, “epidemiology”, “proportion”, “magnitude”, “burden”, “associated factors”, “risk factors”, “predictors”, “determinants”, “healthcare-associated infections”, “healthcare-acquired infections”, and “nosocomial infections”, “hospital acquired infections” and “Ethiopia”. The search strategies were developed using different Boolean operators. Notably, to fit the advanced PubMed database, the following search strategy was applied: [(prevalence) OR incidence[MeSH Terms]) OR epidemiology[MeSH Terms]) OR proportion[MeSH Terms]) OR magnitude[MeSH Terms]) OR burden[MeSH Terms]) AND associated factors) OR risk factors[MeSH Terms]) OR predictors[MeSH Terms]) OR determinants[MeSH Terms]) AND healthcare-associated infections) OR healthcare-acquired infections[MeSH Terms]) OR nosocomial infections[MeSH Terms]) OR hospital acquired infections[MeSH Terms]) AND (Ethiopia)]. Then, we retrieved 611 articles using this PubMed searching strategy.

### Study selection

Duplicate studies were removed using Endnote version 8 (Thomson Reuters, London) reference manager software. The two independent reviewers (AYA and WAB) screened the titles and abstracts. The disagreements were handled based on established article selection criteria. Then, two independent authors (AE and BMB) conducted the abstracts and full-texts review.

### Quality assessment

The two independent authors (DKM and DMB) appraised the quality of the studies. The Joanna Briggs Institute (JBI) quality appraisal checklist was used [15]. The disagreement was resolved by the involvement of a third reviewer (AE). To appraise cohort studies, the following items were used: (i) similarity of groups;(ii) similarity of exposure measurement;(iii) validity and reliability of measurement;(iv) identification of confounder;(v) strategies to deal with confounder;(vi) appropriateness of groups/participants at the start of the study;(vii) validity and reliability of outcome measured;(viii) sufficiency of follow-up time;(ix) completeness of follow-up or descriptions of reason to loss to follow-up;(x) strategies to address incomplete follow-up; and (xi) appropriateness of statistical analysis. The items used to appraise case-control studies were: (i) comparable groups;(ii) appropriateness of cases and controls;(iii) criteria to identify cases and controls;(iv) standard measurement of exposure;(v) similarity in the measurement of exposure for cases and controls; (vi) handling of confounders;(vii) strategies to handle confounder;(viii) standard assessment of outcome;(ix) appropriateness of duration for exposure; and (x) appropriateness of statistical analysis. Cross-sectional studies were appraised based on (i) inclusion criteria;(ii) description of study subject and setting;(iii) valid and reliable measurement of exposure; (iv) the objective and standard criteria used;(v) identification of confounder;(vi) the strategies to handle confounder; (vii) outcome measurement; and (viii) appropriate statistical analysis. All the studies which got 50% and above on the quality assessment scale were considered as low risk.

### Data extraction

Two independent reviewers (AYA and AE) extracted data using a structured data extraction form. Whenever variations of extracted data were observed, the phases were repeated. If discrepancies between data extractors continued, the third reviewer (WAB) was involved. The name of the first author and year, study region, study design, target population, diagnostic methods, sample size, the prevalence of HCAI, and adjusted odds ratio (AOR) of associated factors were collected.

### Outcome measurement

HCAI was considered when reported as infection(s) acquired while receiving medical care based on culture-confirmation [10, 16–18], or clinical and laboratory methods [8, 9, 19–31].

### Statistical analysis

Publication bias was checked visually by the funnel plot, and objectively using Egger’s regression test [32]. Heterogeneity of studies was quantified using the I-squared statistic, in which 25%, 50%, and 75% represented low, moderate, and high heterogeneity, respectively [33]. Pooled analysis was conducted using a weighted-inverse variance random-effects model [34]. The subgroup analysis was done by region, study design, diagnostic method, sample size and ward type. Sensitivity analysis was employed to see the effect of a single study on the overall estimation. Besides, the time-trend analysis was conducted to check the variation through time. STATA version 11 statistical software was used for meta-analysis.

### Ethics approval and consent to participate

Not applicable because no primary data were collected from patients.

## Results

### Literature search

**Fig 1:**
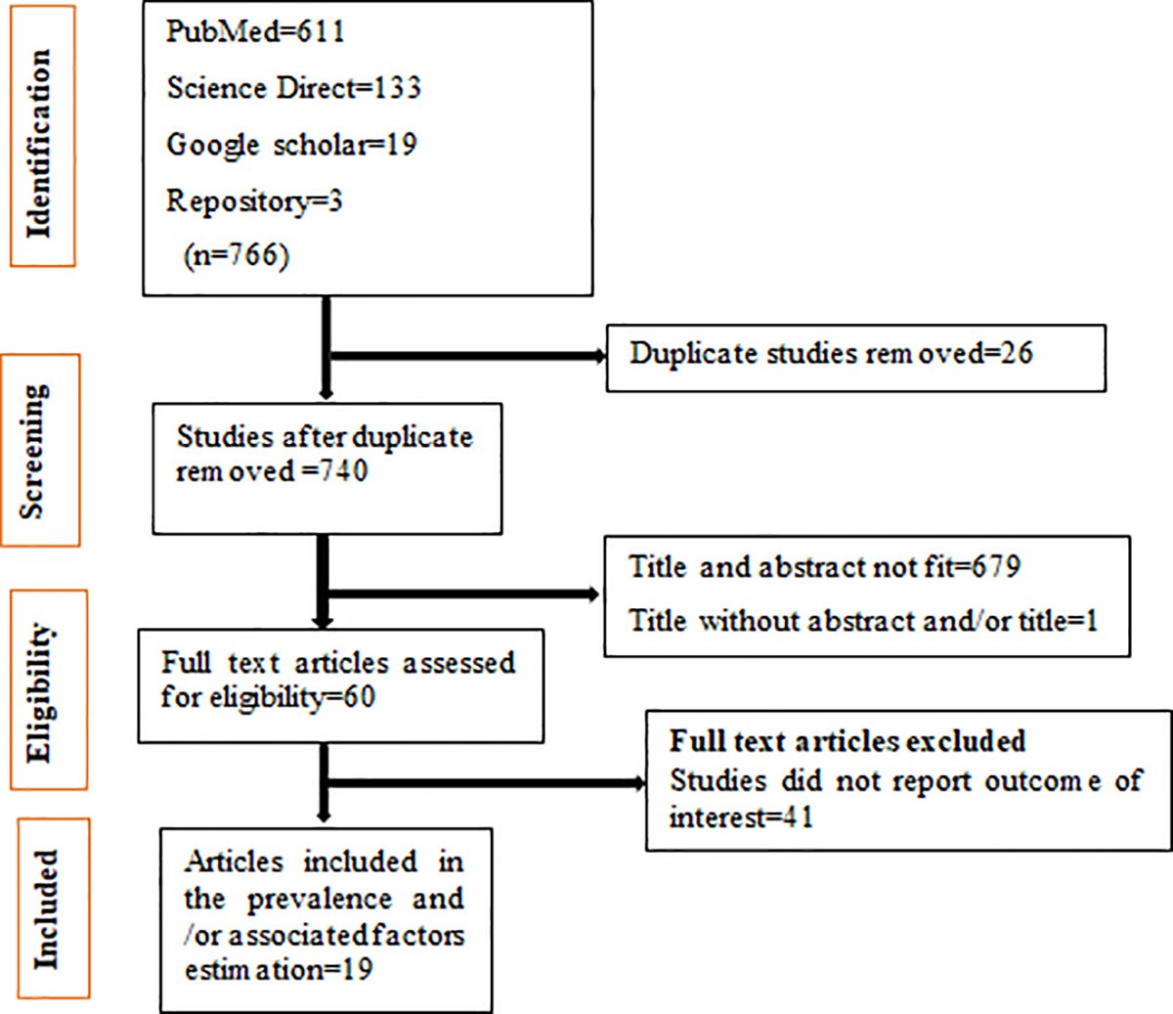
The study selection process.

The search strategy retrieved 611 articles from PubMed, 133 from Science Direct, 19 from Google Scholar, and 3 grey literatures from Addis Ababa University online repository. After duplicates were removed, 740 studies remained. Then, sixty studies were screened for full-text review. Finally, 19 studies were used in the systematic review and/or meta-analysis (**Fig 1**).

### Characteristics of the included studies

Six studies were found in Addis Ababa [19–21, 23, 25, 31], five studies in Amhara region [8, 16, 26, 27, 29], five studies in Oromia [9, 17, 18, 28, 30], one study both in Addis Ababa and Southern Nation Nationalities and People Region (SNNPR) [22], one each in Tigray [24] and SNNPR [10]. Nine studies were conducted across all age groups. Eight studies were done on the adult population and one study was on pediatric patients. Fourteen studies used clinical and laboratory methods for the diagnosis of HCAI while the remaining were culture-confirmed. Four studies were conducted using cohort study design, fourteen were cross-sectional and only one was a case-control study. Only six studies had >1000 sample size (**Table 1**).

**Table 1:** Characteristics and quality status of the studies included.

First author year	Study region	Study design	Sample size	Prevalence	Quality status
Gedebu M. et al./1987 **[19]**	Addis Ababa	Cross-sectional	2506	13.40	Low risk
Gedebu M. et al./1988 **[20]**	Addis Ababa	Cross-sectional	700	17.00	Low risk
Habte-Gaber E. et al./1988 **[21]**	Addis Ababa	Cohort	1006	16.40	Low risk
Berhe N. et al./2001 **[22]**	Addis Ababa and SNNPR	Cohort	247	5.90	Low risk
Endalfer N. et al./2008 **[23]**	Addis Ababa	Cross-sectional	854	9.00	Low risk
Tesfahun Z. et al./2009 **[24]**	Tigray region	Cross-sectional	246	27.60	Low risk
Endalfer N. et al./2011 **[25]**	Addis Ababa	Cross-sectional	215	35.80	Low risk
Melaku S. et al./2012 **[26]**	Amhara region	Cross-sectional	1383	17.80	Low risk
Melaku S. eta al/2012 **[27]**	Amhara region	Cross-sectional	1254	9.40	Low risk
Mulu W. et al./2013 **[16]**	Amhara region	Cross-sectional	294	10.90	Low risk
Sahile T. eta al/2016 **[28]**	Oromia region	Cross-sectional	500	35	Low risk
Yallew WW. et al./2016 **[8]**	Amhara region	Cross-sectional	908	14.90	Low risk
Tolera M. et al./2018 **[18]**	Oromia region	Cross-sectional	394	6.90	Low risk
Gashaw M. et al./2018 **[17]**	Oromia region	Cross-sectional	1015	11.60	Low risk
Ali S. et al./2018 **[9]**	Oromia region	Cohort	1069	19.40	Low risk
Alemayehu T. et al./2019 **[10]**	SNNPR	Cross-sectional	939	21.40	Low risk
Gebremeskel S. et al./2018 **[31]**	Addis Ababa	Cross-sectional	410	19.80	Low risk
Yallew WW. et al./2017 **[29]**	Amhara region	Case-control	545		Low risk
Zewdu et al./2017 **[30]**	Oromia region	Cohort	300	14.00	Low risk

**Note:** SNNPR: Southern Nations Nationalities and Peoples Region; Low risk: a study scored > 50% in the JBI quality assessment scale.

### Quality of studies

The JBI quality appraisal criteria established for cross-sectional, case-control, and cohort studies were used. The studies included in this systematic review and meta-analysis had no considerable risk. Therefore, all the studies were considered [8–10, 16–31] (**Table 1**).

### Meta-analysis

#### Publication bias

**Fig 2:**
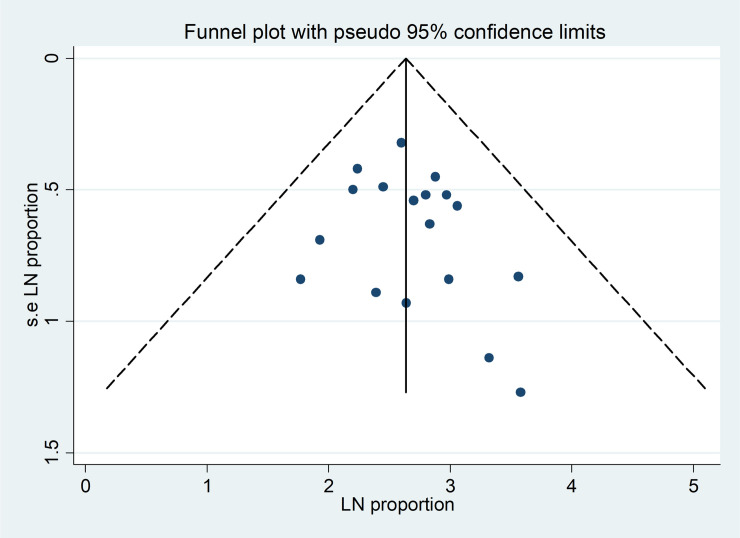
Funnel plot for publication bias, LN of proportion (X-axis) with its standard error of LN of proportion (Y-axis).

The funnel plot showed symmetrical distribution (**Fig 2**). Egger’s regression test p-value was 0.328, which indicated the absence of publication bias.

#### The prevalence of healthcare-associated infection

**Fig 3:**
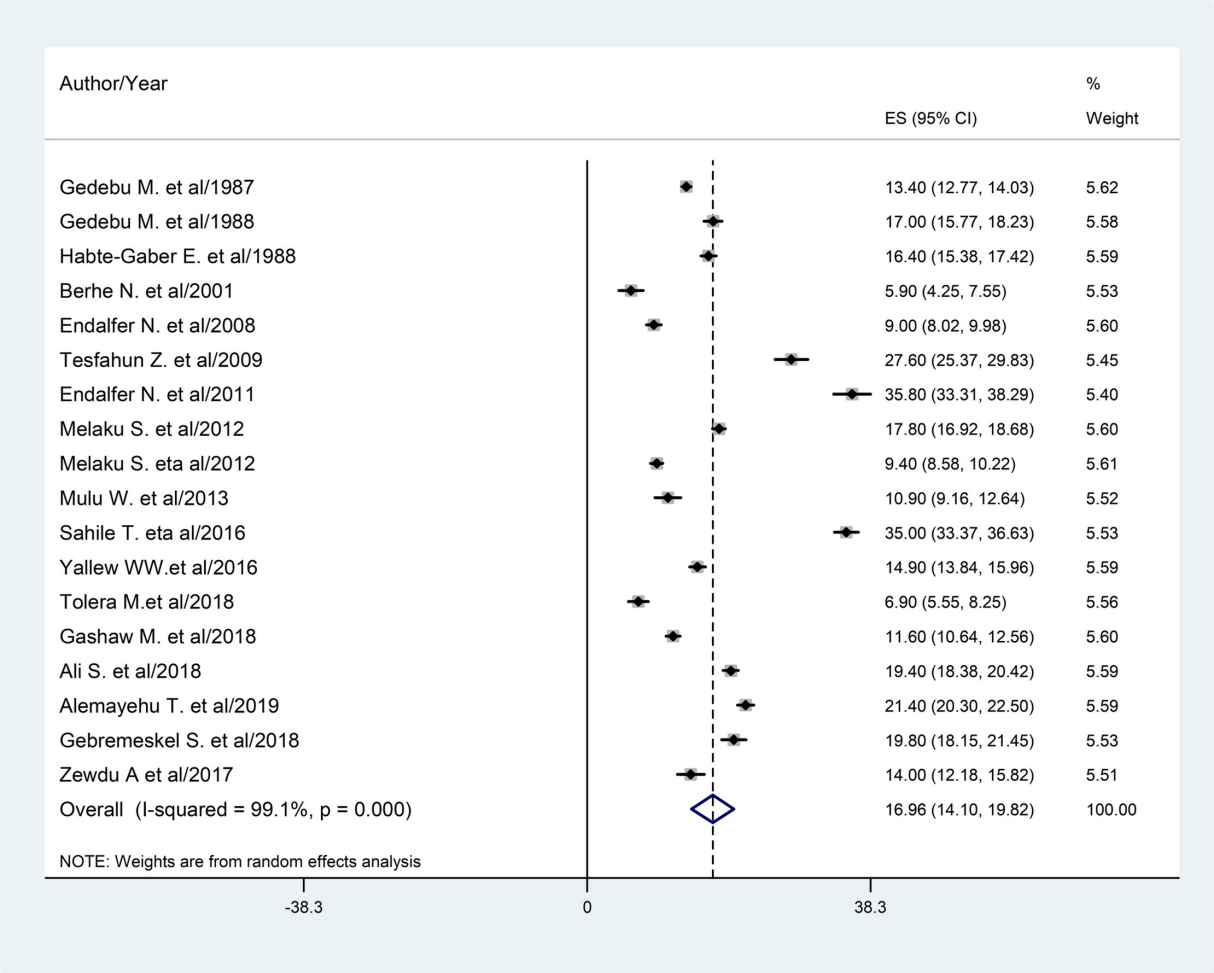
Forest plot of the pooled prevalence (ES) of HCAI with corresponding 95% CIs.

*A total of 18 studies were used and 14*,*240 patients participated in the* prevalence estimation. The estimated overall prevalence of HCAI is presented in a forest plot (**Fig 3**). The overall prevalence of HCAI was 16.96% (95% confidence interval (CI): 14.10%-19.82%).

#### Subgroup analysis

The subgroup analyses based on study region, study design, diagnostic method, and the sample size were done. Accordingly, the prevalence of HCAI was found 27.6% in Tigray region, 18.2% diagnosed by clinical and laboratory methods, 17.83% in the cross-sectional studies, 18.15% in studies using < 1000 study samples (**Table 2**).

**Table 2:** The pooled prevalence of HCAI, 95% CI, and heterogeneity estimate with a p-value for the subgroup analysis, by region, study design, sample size, and diagnostic method.

Variables	Characteristics	Pooled prevalence (95% CI)	I^2^
**Region**	Addis Ababa	18.44% (14.02–22.86)	99%
	Oromia	17.37% (9.2–25.56)	99.5%
	Amhara	13.27% (9.00–17.52)	98.5%
	Tigray	27.6% (25.37–29.83)	-
	SNNPR	21.4% (20.3–22.5)	-
	Addis Ababa & SNNPR	5.9% (4.25–7.55)	-
**Study design**	Cross-sectional	17.83% (14.39–21.27)	99.3%
	Cohort	13.96% (8.78–19.14)	98.4%
**Diagnostic method**	Clinical and laboratory	18.2% (14.85–21.51)	99.2%
	Culture-confirmed	12.71% (6.4–19.02)	99%
**Sample size**	<1000	18.15% (13.28–23.03)	99.3%
	≥1000	14.66% (11.72–17.59)	98.6%

**Note:** SNNPR: Southern Nations Nationalities and Peoples Region; I^2^: reported for the pooled effect of two or more studies.

The prevalence of HCAI was reported across various wards too. A study conducted at Jimma University Hospital showed that the incidence of HCAI was the highest in the Intensive Care Unit (ICU) (207.55/1000 patient-days) followed by the pediatric ward (69.16/1000 patient-days), and surgical ward (28.87/1000 patient-days) [9]. In two studies, Yallew WW. et al. [8] and Ali S. et al. [9], HCAI was the lowest in the ophthalmology ward.

**Fig 4:**
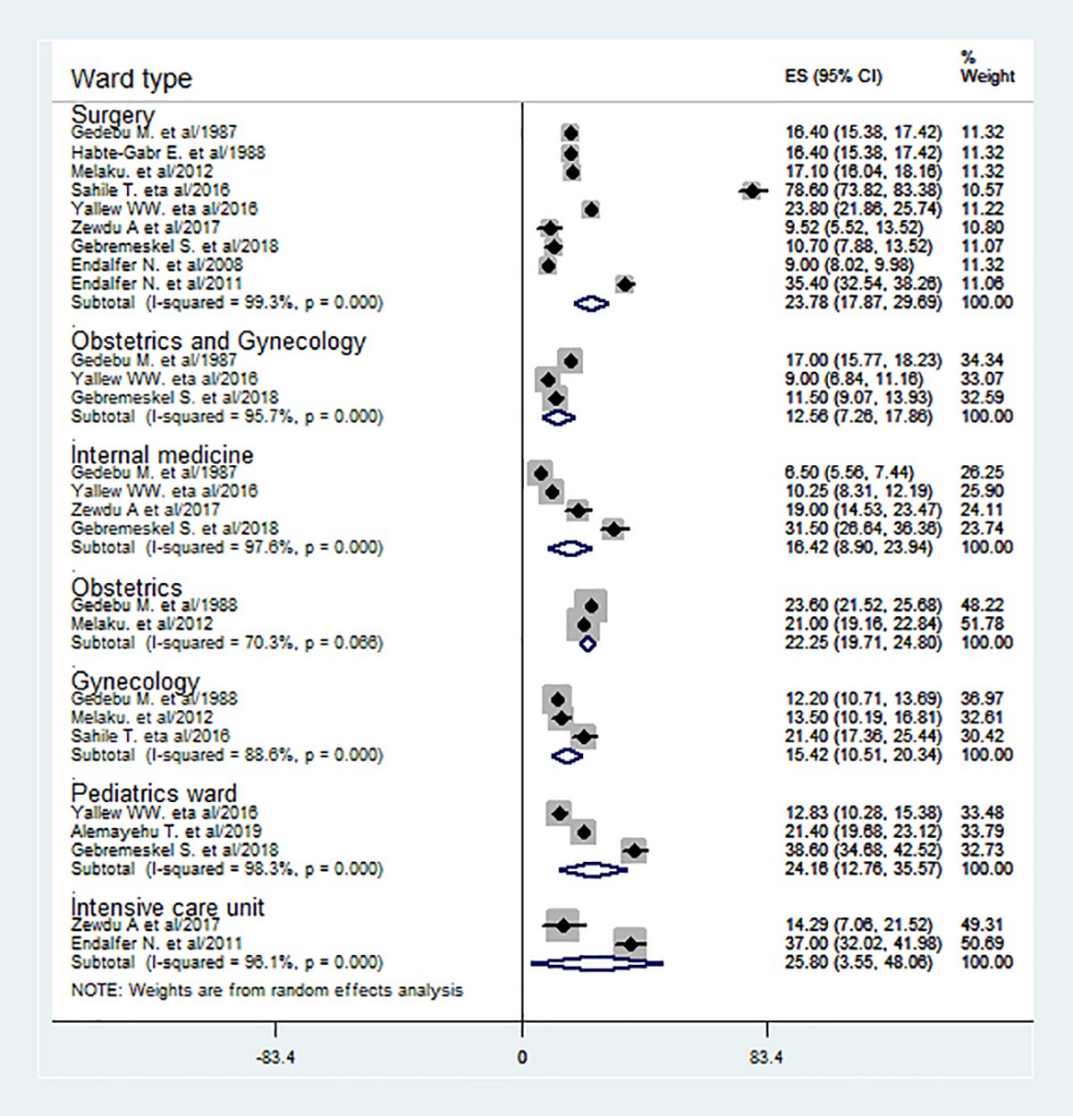
The prevalence of HCAI is based on the subgroup analysis by ward type with corresponding 95% CIs.

Besides, in this meta-analysis, HCAI was estimated in different wards based on the pooled effect of two or more studies. As estimated from the effect of two studies [25, 30], the prevalence of HCAI was the highest in ICU (25.8%) followed by pediatrics (24.16%) [8, 10, 31], surgical (23.78%) [8, 19, 21, 23, 25, 26, 28, 30, 31] and obstetrics ward (22.25%) [19, 26] (**Fig 4**).

#### Sensitivity analysis

**Fig 5:**
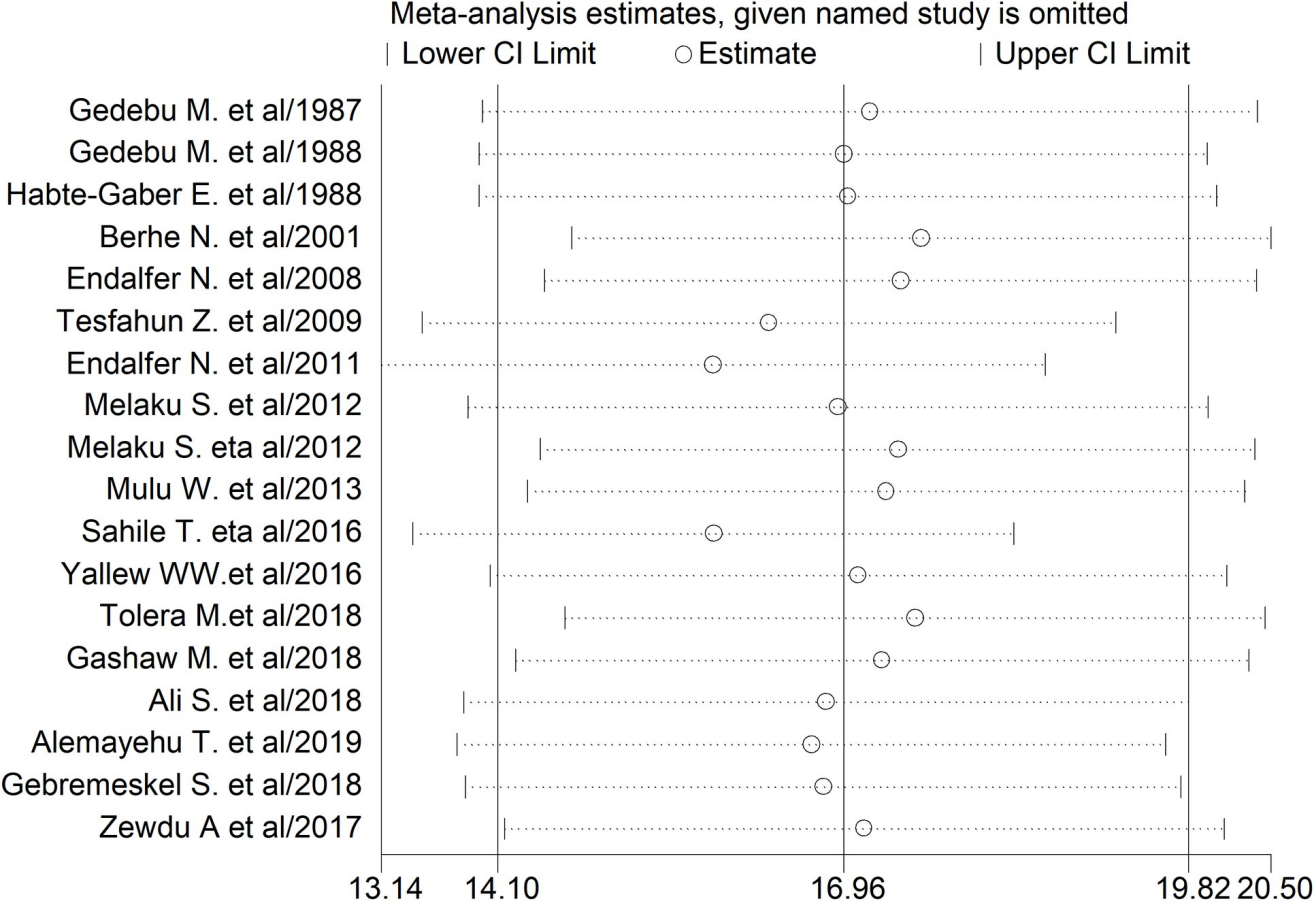
The sensitivity analysis showed the pooled prevalence when the studies were omitted step by step.

The studies of Endalafer N. et al. [25] and Sahile T. et al. [28] had shown an impact on the overall estimate of HCAI (**Fig 5**).

#### Time-trend analysis

**Fig 6:**
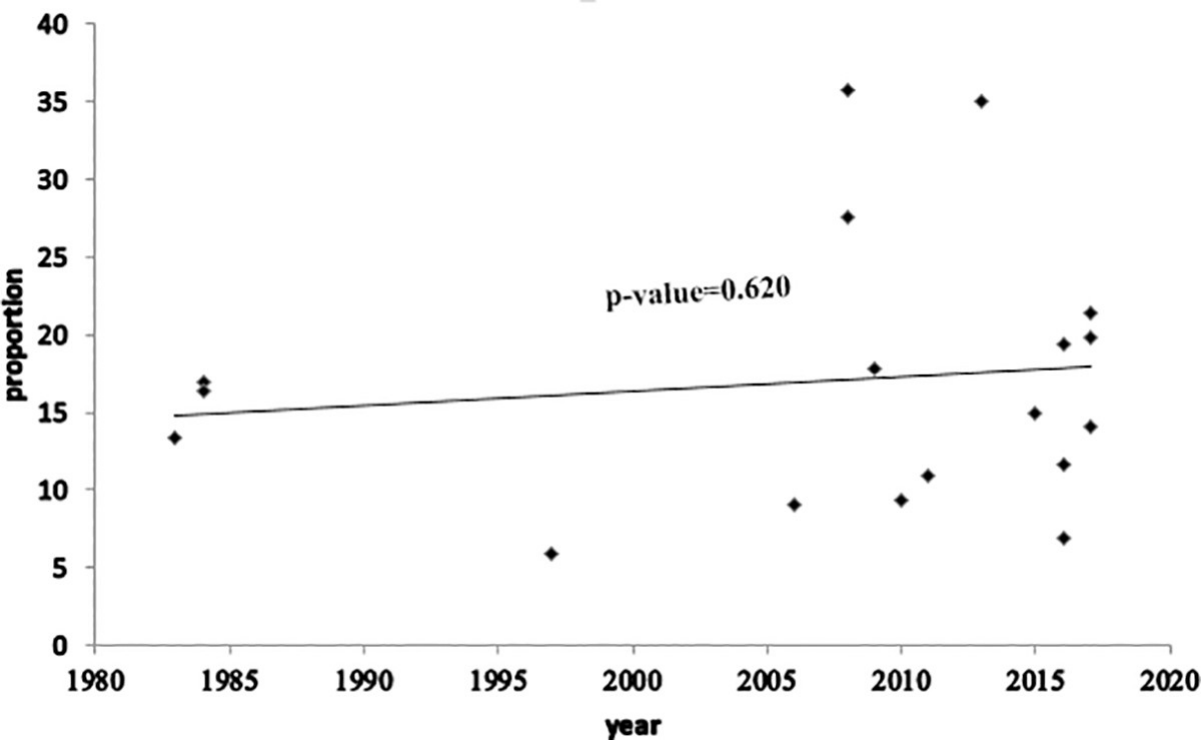
Time-trend analysis of the prevalence of HCAI in Ethiopia from 1983 to 2017.

The time-trend analysis showed that the prevalence of HCAI was increased from 13.4% in 1983 to 19.8% in 2017. However, the pooled prevalence was not increasing significantly from year to year (p-value: 0.620) (**Fig 6**).

#### Determinants of healthcare-associated infection

In this systematic review and meta-analysis, HCAI in the Ethiopian context is associated with socio-demographic, patient health condition, and healthcare-related risk factors. Thus, based on the report of a single study, the age range of the patient 18–30 years was found to be protective (AOR = 0.54; 95% CI: 0.22–0.85) [9] (**Table 3**). On the contrary, based on the reports of individual studies included, HCAI had shown a positive association with the following healthcare-related factors: taking prophylaxis (AOR = 1.76; 95% CI: 1.21–2.3) [27]; admission to the surgical ward (AOR = 2.86; 95 CI: 1.33–4.38) [8]; admission at Felege Hiwot Referal Hospital (FHRH) (AOR = 1.99; 95% CI: 1.2–2.77) [8] and chest tube insertion (AOR = 4.14; 95% CI: 1.57–6.71) [9].

**Table 3:** Determinants of healthcare-associated infection in Ethiopia.

Determinants	Author/year	HCAI		Effect size (95% CI)	Pooled effect size (95% CI)	*I*^*2*^
		Yes	No			
**Surgical procedure**	Endalfer N. eta al/2011 [25]	71	66	3.96 (2.82–5.09)	3.37 (1.85–4.89)	46.9%
	Yallew WW. et al./2017 [29]	64	164	2.35 (0.35–4.34)		
**Take prophylaxis**	Melaku S. et al./2012 [27]	54	237	1.76 (1.21–2.3)	1.76 (1.21–2.3)	-
**underlying non-communicable disease**	Melaku S. et al./2012 [27]	16	71	4.3 (2.32–6.28)	2.81 (1.39–4.22)	54.5%
	Mulu W. et al./2013 [16]	10	36	2.72 (0.42–5.01)		
	Ali S. et al./2018 [9]	44	135	2.01 (1.15–2.87)		
**Age ≥ 51 years**	Mulu W. et al./2013 [16]	6	16	6.38 (-10.61–23.37)	6.38 (-10.61–23.37)	-
**Duration of operation 90–150 minutes**	Mulu W. et al./2013 [16]	3	6	11 (-18.41–40.41)	11 (-18.41–40.41)	-
**Hospital stay >5 days**	Mulu W. et al./2013 [16]	2	3	8.2 (5.2–11.2)	5.32 (0.01–10.65)	89.8%
	Alemayehu T. et al./2019 [10]	58	183	2.76 (1.13–4.37)		
**Age 1–14 years**	Yallew WW. et al./2016 [8]	14	148	0.25 (-0.06–0.56)	0.25 (-0.06–0.56)	-
**Admission to the surgery ward**	Yallew WW. et al./2016 [8]	75	240	2.86 (1.33–4.38)	2.86 (1.33–4.38)	-
**Patients admitted at Felege Hiwot Hospital**	Yallew WW. et al./2016 [8]	74	261	1.99 (1.2–2.77)	1.99 (1.2–2.77)	-
**Immuno-deficiency**	Yallew WW. et al./2017 [29]	31	92	2.34 (0.57–4.1)	2.34 (0.57–4.1)	-
**Central vascular catheter**	Yallew WW. et al./2017 [29]	5	4	6.92 (-11.17–25.01)	6.92 (-11.17–25.01)	-
**Patient received antimicrobial**	Yallew WW. et al./2017 [29]	104	294	8.63 (-1.79–19.05)	8.63 (-1.79–19.05)	-
**Medical waste container at room**	Yallew WW. et al./2017 [29]	102	431	0.18 (-0.290–0.65)	0.18 (-0.290–0.65)	-
**Previous hospitalization**	Ali S. et al./2018 [29]	20	25	1.65 (0.91–2.39)	2.13 (0.71–3.55)	49.4%
	Gebremeskel S. et al./2018 [31]	27	43	3.22 (1.16–5.28)		
**Age 18–30 years**	Ali S. et al./2018 [9]	28	179	0.54 (0.22–0.85)	0.54 (0.22–0.85)	-
**Chest tube insertion**	Ali S. et al./2018 [9]	3	1	4.14 (1.57–6.71)	4.14 (1.57–6.71)	-
**Mechanical ventilation**	Ali S. et al./2018 [9]	12	22	1.99 (0.65–3.32)	1.99 (0.65–3.32)	-
**Malnutrition**	Alemayehu T. et al./2019 [10]	39	135	2.1 (0.78–3.41)	2.1 (0.78–3.41)	-
**Male sex**	Gebremeskel S. et al./2018 [31]	38	95	2.1 (0.45–3.67)	2.1 (0.45–3.67)	-
**Hospital stay < 5 days**	Gebremeskel S. et al./2018 [31]	5	119	0.03 (-0.01–0.07)	0.03 (-0.01–0.07)	-

**Note:** I^2^: reported for the pooled effect of two or more studies.

Moreover, in this meta-analysis, the determinants of HCAI were identified based on the pooled effect of two or more studies. Hence, as estimated from the pooled effect of two studies [25, 29], HCAI was 3.37 times (AOR = 3.37; 95% CI: 1.85–4.89) more likely among patients who had the surgical procedure as compared to no surgical procedure. Similarly, based on the pooled effect of three studies [9, 16, 27], patients who had underlying non-communicable disease were 2.81 times more likely to have HCAI as compared to those without the underlying disease (**Table 3**).

## Discussion

In this systematic review and meta-analysis, the pooled prevalence of HCAI was 16.96% in Ethiopia. The authors also found that surgical procedures and underlying non-communicable diseases were identified as determinants of HCAI.

From the study, the national pooled prevalence of HCAI in Ethiopia was 16.96% (95% CI: 14.10%-19.82%). The result was higher than studies conducted in China (3.12%) [35], Morocco (10.3%) [36], Botswana (13.54%) [6], and South Africa (7.67%) [7]. The possible reasons for high prevalence in this study might be very low hand hygiene practice by physicians and resource constraints [37], low adherence to infection prevention practice [38], low level of job satisfaction [39], morally distressed nurses [40], and low implementation of the nursing process [41] in our settings, and also less attention given to HCAI.

Regarding hand hygiene, only 7% of the physicians working at two University hospitals in the capital of Ethiopia, Addis Ababa, performed hand hygiene before patient contact [37]. As a result, the acquisition of HCAI from these healthcare professionals might be high. Evidence also showed that 35% of the nurses in southwest Ethiopia were non-adherent to infection prevention practice [38], thereby contributing to high HCAI in Ethiopia. Besides, nearly 68% of the health professionals were less satisfied with their work in one of the regions in the country [39]. Hence, the nosocomial infection becomes inevitably high because these less satisfied health professionals are less likely to deliver quality healthcare. Additionally, about 84% of the nurses in the northwestern part of the country [40] were morally distressed, thus causing HCAI as morally less prepared nurses were unable to deliver quality nursing care.

Resource constraints could also increase HCAI in the country because lack of hand hygiene agents and sinks were reported as hindering factors of infection prevention practice in Addis Ababa, Ethiopia [37]. Implementation of the nursing process was below half (49%) in the northwest part of the country [41], so nursing intervention would not be planed for patients at risk of nosocomial infection. Furthermore, healthcare providers, patients, and/or families are more curious about the primary reason for admission or healthcare visits, so less attention is given to HCAI.

From the subgroup analysis, HCAI was found the highest in ICU (26%). This finding is consistent with studies conducted in China [42], India [43], and Morocco [36]. The reasons for high HCAI in ICU may be due to the highest incidence of HCAI, the severity of the disease, and highly invasive procedures. The incidence of HCAI at a referral medical center in Jimma University Hospital, Ethiopia was 207.6/1000 patient-days [9]. This highest incidence may be augmented by the severity of the disease [36] among ICU patients. Added, highly invasive procedures like intubation, peripheral, and central venous catheters are highly likely among ICU patients. Consequently, the risk of HCAI is higher among intubated patients and those on vascular catheterization [44].

In the time trend analysis, we found that HCAI was slightly increasing in Ethiopia from 1983 to 2017. The possible reasons might be more emphasis given on healthcare coverage than quality, increase in technological advancement, and overutilization of invasive procedures. Evidence revealed that advances in life-saving medical practices increase exposure to invasive procedures which increase the occurrences of nosocomial infections [11]. On top of this, nurses’ burnout might contribute to the increasing trend. Evidence in the United States (US) revealed that nurses’ burnout was found as a single most important associated factor for increased nosocomial urinary tract infection (UTI) and surgical site infection (SSI) [45].

The current systematic review and meta-analysis revealed surgical procedure and underlying non-communicable disease as determinants of HCAI. Accordingly, patients who had the surgical procedure were 3.37 times more likely to acquire HCAI as compared to patients who have no surgical procedure. The finding is in line with previous studies done in South Africa [7] and Poland [44]. The reason for the observed association could be explained by less compliance to hand hygiene practice and high prevalence of surgical site infection (25.22%) in Ethiopia [46]. Compliance with hand hygiene practice is pivotal for the prevention and control of nosocomial infection, but only 3.6% keep hand hygiene before performing aseptic procedures at Debre Birhan referral hospital, central Ethiopia [47]. In 2017, the overall compliance to hand hygiene practice was 18.7% [48] and 22% [47] at Hiwot Fana Specialized Hospital and Debre Birhan Referral Hospital in Ethiopia, respectively. Consequently, unable to keep and maintain hand hygiene practice increased the acquisition of HCAI.

In this systematic review and meta-analysis, the odds of having HCAI among patients who have underlying non-communicable disease were nearly 3 times higher than their counter-part. This finding is supported by studies that reported positive association of HCAI with diabetes mellitus [49] and underlying renal disease [50]. The possible explanations for the observed association in the current study might be due to high prevalence of underlying diseases and the immune-suppressive effects of these diseases. In Ethiopia, a meta-analysis of studies showed high burden (6.5%) of diabetes mellitus [51]. Besides, another meta-analysis in other setting (North America, Europe, Latin America, and the Caribbean) reported immune-suppression as risk factor for HCAI [49]. Thus, higher odds of HCAI may be ascertained to the aforementioned antecedents.

## Strengths and limitations of the study

This systematic review and meta-analysis was the first national report on the prevalence of HCAI and its determinants in Ethiopia. However, it may lack national representativeness because no data were found from Benishangul Gumuz, Afar, Gambella, Somalia, Dire Dawa, and Harari regions of the country. Besides, the use of only English language, the absence of grey pieces of literature, and the unlimited time-period for the inclusion of studies may limit the conclusiveness of the finding. On top of this, the time-trend analysis might not reflect the exact trend because all the considered years didn’t have reported data.

## Conclusions

The prevalence of healthcare-associated infection has remained a problem of public health importance in Ethiopia. Based on the subgroup analysis, the highest prevalence of HCAI was found in ICU followed by pediatrics, surgical, and obstetrics wards in descending order. Surgical procedures and underlying non-communicable disease were found as determinants of HCAI. Therefore, policy-makers and program officers should give due emphasis to the prevention of healthcare-associated infection with more attention for patients admitted to ICU. Furthermore, the existing infection prevention and control practices for patients who had surgical procedures and underlying non-communicable disease should be strengthened in Ethiopia.

## Supporting information

S1 File PRISMA checklist. (DOC)

## Abbreviations

AOR: Adjusted Odds Ratio
CI: Confidence Interval
HCAI: Healthcare-Associated Infection
ICU: Intensive Care Unit
SNNPR: Sothern Nations Nationalities and Peoples Region
WHO: World Health Organization

## References

[1] Garner JS, Jan WR, Emori TG, Horan TC, Hughes JM. CDC definitions for nosocomial infections, 1988. American Journal of INFECTIONCONTROL. 1988;16(3). 10.1016/0196-6553(88)90053-3 2841893

[2] Horan TC, Andrus M, Dudeck MA. CDC/NHSN surveillance definition of health care–associated infection and criteria for specific types of infections in the acute care setting. American journal of infection control. 2008;36(5):309–32. 10.1016/j.ajic.2008.03.002 18538699

[3] Health care-associated infections FACT SHEET [Internet]. [cited 02 Feb 2020]. Available from: https://www.who.int/gpsc/country_work/gpsc_ccisc_fact_sheet_en.pdf.

[4] World Health Organization. Report on the Burden of Endemic Health Care-Associated Infection Worldwide. Geneva, Switzerland: WHO Document Production Services; 2011.

[5] Rothe C, Schlaich C, Thompson S. Healthcare-associated infections in sub-Saharan Africa. Journal of Hospital Infection. 2013;85(4):257–67. 10.1016/j.jhin.2013.09.008 24209885

[6] Mpinda-Joseph P, Anand Paramadhas BD, Reyes G, Maruatona MB, Chise M, Monokwane-Thupiso BB, et al Healthcare-associated infections including neonatal bloodstream infections in a leading tertiary hospital in Botswana. Hospital Practice. 2019;47(4):203–10. 10.1080/21548331.2019.1650608 31359809

[7] Nair A, Steinberg W, Habib T, Saeed H, Raubenheimer J. Prevalence of healthcare-associated infection at a tertiary hospital in the Northern Cape Province, South Africa. South African Family Practice. 2018;60(5):162–7.

[8] Yallew WW, Kumie A, Yehuala FM. Point prevalence of hospital-acquired infections in two teaching hospitals of Amhara region in Ethiopia. Drug, healthcare and patient safety. 2016;8:71 10.2147/DHPS.S107344 27601932 PMC5003516

[9] Ali S, Birhane M, Bekele S, Kibru G, Teshager L, Yilma Y, et al Healthcare associated infection and its risk factors among patients admitted to a tertiary hospital in Ethiopia: longitudinal study. Antimicrobial Resistance & Infection Control. 2018;7(1):2. 29312659 10.1186/s13756-017-0298-5 PMC5755436

[10] Alemayehu T, Tadesse E, Ayalew S, Nigusse B, Yeshitila B, Amsalu A, et al High burden of nosocomial infections caused by multi-drug re-sistant pathogens in pediatric patients at hawassa university comprehensive specialized hospital. 2019.

[11] Bereket W, Hemalatha K, Getenet B, Wondwossen T, Solomon A, Zeynudin A, et al Update on bacterial nosocomial infections. European review for medical and pharmacological sciences. 2012;16(8):1039–44. 22913154

[12] Schumacher M, Wangler M, Wolkewitz M, Beyersmann J. Attributable mortality due to nosocomial infections. Methods of information in medicine. 2007;46(05):595–600. 17938785

[13] Allegranzi B, Storr J, Dziekan G, Leotsakos A, Donaldson L, Pittet D. The first global patient safety challenge “clean care is safer care”: from launch to current progress and achievements1. Journal of Hospital Infection. 2007;65:115–23. 10.1016/S0195-6701(07)60027-9 17540254

[14] Moher D, Liberati A, Tetzlaff J, Altman DG, Group P. Preferred reporting items for systematic reviews and meta-analyses: the PRISMA statement. PLoS med. 2009;6(7):e1000097 10.1371/journal.pmed.1000097 19621072 PMC2707599

[15] Peters MD, Godfrey CM, McInerney P, Soares CB, Khalil H, Parker D. The Joanna Briggs Institute reviewers' manual 2015: methodology for JBI scoping reviews 2015 Available from: https://www.who.int/nutrition/publications/globaltargets2025_policybrief_lbw/en/.

[16] Mulu W, Kibru G, Beyene G, Damtie H. Associated risk factors for postoperative nosocomial infections among patients admitted at Felege Hiwot Referral Hospital, Bahir Dar, Northwest Ethiopia. Clinical medicine & research. 2013;2(6):140–7.

[17] Gashaw M, Berhane M, Bekele S, Kibru G, Teshager L, Yilma Y, et al Emergence of high drug resistant bacterial isolates from patients with health care associated infections at Jimma University medical center: a cross sectional study. Antimicrobial resistance and infection control. 2018;7:138 10.1186/s13756-018-0431-0 30479751 PMC6245755

[18] Tolera M, Abate D, Dheresa M, Marami D. Bacterial nosocomial infections and antimicrobial susceptibility pattern among patients admitted at Hiwot Fana Specialized University Hospital, Eastern Ethiopia. Advances in medicine. 2018;2018. 10.1155/2018/2127814 PMC6305041 30631777

[19] Gedebou M, Kronvall G, Habte-Gabr E, Ringertz S. The bacteriology of nosocomial infections at Tikur Anbessa Teaching Hospital, Addis Ababa. Acta pathologica, microbiologica, et immunologica Scandinavica Section B, Microbiology. 1987;95(6):331–6. 10.1111/j.1699-0463.1987.tb03134.x 3439462

[20] Gedebou M, Habte-Gabr E, Kronvall G, Yoseph S. Hospital-acquired infections among obstetric and gynaecological patients at Tikur Anbessa Hospital, Addis Ababa. The Journal of hospital infection. 1988;11(1):50–9. 10.1016/0195-6701(88)90039-4 2895138

[21] Habte-Gabr E, Gedebou M, Kronvall G. Hospital-acquired infections among surgical patients in Tikur Anbessa Hospital, Addis Ababa, Ethiopia. Am J Infect Control. 1988;16(1):7–13. 10.1016/0196-6553(88)90004-1 3369748

[22] Berhe N, Hailu A, Abraham Y, Tadesse Y, Breivik K, Abebe Y. Inter-current and nosocomial infections among visceral leishmaniasis patients in Ethiopia: an observational study. Acta tropica. 2001;80(2):87–95. 10.1016/s0001-706x(01)00156-5 11600084

[23] Endalafer N. Bacterial Nosocomial Infections and Their Antimicrobial Susceptibility Patterns in Surgical Wards and Surgical Intensive Care Unit (SICU) Of Tikur Anbessa University Hospital Addis Ababa, Ethiopia: Addis Ababa University; 2008.

[24] Tesfahunegn Z, Asrat D, Woldeamanuel Y, Estifanos K. Bacteriology of surgical site and catheter related urinary tract infections among patients admitted in Mekelle Hospital, Mekelle, Tigray, Ethiopia. Ethiop Med J. 2009;47(2):117–27. 19743791

[25] Endalafer N, Gebre-Selassie S, Kotiso B. Nosocomial bacterial infections in a tertiary hospital in Ethiopia. Journal of Infection Prevention. 2011;12(1):38–43.

[26] Melaku S, Gebre-Selassie S, Damtie M, Alamrew K. Hospital acquired infections among surgical, gynaecology and obstetrics patients in Felege-Hiwot referral hospital, Bahir Dar, northwest Ethiopia. Ethiop Med J. 2012;50(2):135–44. 22924282

[27] Melaku S, Kibret M, Abera B, Gebre-Sellassie S. Antibiogram of nosocomial urinary tract infections in Felege Hiwot referral hospital, Ethiopia. Afr Health Sci. 2012;12(2):134–9. 10.4314/ahs.v12i2.9 23056018 PMC3462551

[28] Sahile T, Esseye S, Beyene G, Ali SJIJTDH. Post-surgical infection and antibiotic susceptibility patterns of bacteria isolated from admitted patients with signs of infection at Jimma University specialized hospital, Jimma, Ethiopia. 2016;17(4):1–12.

[29] Yallew WW, Kumie A, Yehuala FM. Risk factors for hospital-acquired infections in teaching hospitals of Amhara regional state, Ethiopia: A matched-case control study. PLoS One. 2017;12(7):e0181145 10.1371/journal.pone.0181145 28719665 PMC5515417

[30] Zewdu A. Health Care Associated Infection at Adama Hospital Medical College, Adama Oromia, Ethiopia. Addis Ababa: Addis Ababa University 2017.

[31] Gebremeskel S. Management of Hospital-Acquired Infections among Hospitalized Patients at Zewditu Memorial Hospital, Addis Ababa, Ethiopia: A Prospective Study: Addis Ababa Universty; 2018.

[32] Rothstein HR, Sutton AJ, Borenstein M. Publication bias in meta-analysis. 2005.

[33] Ioannidis JP. Interpretation of tests of heterogeneity and bias in meta‐analysis. Journal of evaluation in clinical practice. 2008;14(5):951–7. 10.1111/j.1365-2753.2008.00986.x 19018930

[34] Borenstein M, Hedges LV, Higgins JP, Rothstein HR. A basic introduction to fixed‐effect and random‐effects models for meta‐analysis. Research synthesis methods. 2010;1(2):97–111. 10.1002/jrsm.12 26061376

[35] Wang J, Liu F, Tartari E, Huang J, Harbarth S, Pittet D, et al The prevalence of healthcare-associated infections in mainland China: a systematic review and meta-analysis. 2018;39(6):701–9. 10.1017/ice.2018.60 29655388

[36] Razine R, Azzouzi A, Barkat A, Khoudri I, Hassouni F, Chefchaouni AC, et al Prevalence of hospital-acquired infections in the university medical center of Rabat, Morocco. International archives of medicine. 2012;5(1):26 10.1186/1755-7682-5-26 23031793 PMC3515421

[37] Tenna A, Stenehjem EA, Margoles L, Kacha E, Blumberg HM, Kempker RRJIC, et al Infection control knowledge, attitudes, and practices among healthcare workers in Addis Ababa, Ethiopia. 2013;34(12):1289–96. 10.1086/673979 PMC3995333 24225614

[38] Bekele I, Yimam I, Akele GJIR. Adherence to Infection prevention and factors among nurses in jimma university medical center. 2018;14(2):1–7.

[39] Temesgen K, Aycheh MW, Leshargie CTJH, outcomes qol. Job satisfaction and associated factors among health professionals working at Western Amhara Region, Ethiopia. 2018;16(1):65. 10.1186/s12955-018-0898-7 PMC5905160 29665856

[40] Berhie AY, Tezera ZB, Azagew AWJPR, Management B. Moral Distress and Its Associated Factors Among Nurses in Northwest Amhara Regional State Referral Hospitals, Northwest Ethiopia. 2020;13:161. 10.2147/PRBM.S234446 PMC7037049 32110124

[41] Semachew A. Implementation of nursing process in clinical settings: the case of three governmental hospitals in Ethiopia, 2017. BMC research notes. 2018;11(1):173 10.1186/s13104-018-3275-z 29534756 PMC5851090

[42] Liu J, Wu Y, Cai M, Zhou C. Point-prevalence survey of healthcare-associated infections in Beijing, China: a survey and analysis in 2014. Journal of Hospital Infection. 2016;93(3):271–9. 10.1016/j.jhin.2016.03.019 27140419

[43] Nair V, Sahni AK, Sharma D, Grover N, Shankar S, Chakravarty A, et al Point prevalence & risk factor assessment for hospital-acquired infections in a tertiary care hospital in Pune, India. The Indian journal of medical research. 2017;145(6):824–32. 10.4103/ijmr.IJMR_1167_15 29067985 PMC5674553

[44] Deptuła A, Trejnowska E, Ozorowski T, Hryniewicz WJJoHI. Risk factors for healthcare-associated infection in light of two years of experience with the ECDC point prevalence survey of healthcare-associated infection and antimicrobial use in Poland. 2015;90(4):310–5. 10.1016/j.jhin.2015.03.005 25933918

[45] Cimiotti JP, Aiken LH, Sloane DM, Wu ES. Nurse staffing, burnout, and health care–associated infection. American Journal of Infection Control. 2012;40(6):486–90. 10.1016/j.ajic.2012.02.029 22854376 PMC3509207

[46] Birhanu Y, Endalamaw A. Surgical site infection and pathogens in Ethiopia: a systematic review and meta-analysis. Patient Safety in Surgery. 2020;14(1):1–8. 32110246 10.1186/s13037-020-00232-y PMC7035652

[47] Kolola T, Gezahegn T. A twenty-four-hour observational study of hand hygiene compliance among health-care workers in Debre Berhan referral hospital, Ethiopia. Antimicrobial resistance and infection control. 2017;6(1):109. 29093813 10.1186/s13756-017-0268-y PMC5663127

[48] Awoke N, Geda B, Arba A, Tekalign T, Paulos KNr, practice. Nurses Practice of Hand Hygiene in Hiwot Fana Specialized University Hospital, Harari Regional State, Eastern Ethiopia: Observational Study. Nursing research. 2018;2018. 10.1155/2018/2654947 PMC5926477 29850239

[49] Rodríguez-Acelas AL, de Abreu Almeida M, Engelman B, Cañon-Montañez W. Risk factors for health care–associated infection in hospitalized adults: Systematic review and meta-analysis. American Journal of Infection Control. 2017;45(12):e149–e56. 10.1016/j.ajic.2017.08.016 29031433

[50] Greene MT, Chang R, Kuhn L, Rogers MA, Chenoweth CE, Shuman E, et al Predictors of hospital-acquired urinary tract–related bloodstream infection. 2012;33(10):1001–7. 10.1086/667731 PMC3442945 22961019

[51] Bishu KG, Jenkins C, Yebyo HG, Atsbha M, Wubayehu T, Gebregziabher M. Diabetes in Ethiopia: A systematic review of prevalence, risk factors, complications, and cost. Obesity Medicine. 2019;15:100132.

