# Supplementary material for: Healthcare-associated infection and its determinants in Ethiopia: A systematic review and meta-analysis
Source: PLoS One. 2020 Oct 23;15(10):e0241073. doi: 10.1371/journal.pone.0241073 (PMC7584210; doi:10.1371/journal.pone.0241073)
Supplement: Attachment — Submitted filename: PONE-D-20-09587_reviewer submission.pdf [file pone.0241073.s002.pdf]

PLOS ONE

Prevalence and risk factors of healthcare-associated infection in Ethiopia: a systematic review and meta-analysis

--Manuscript Draft--

**Manuscript Number:**	PONE-D-20-09587
**Article Type:**	Research Article
**Full Title:**	Prevalence and risk factors of healthcare-associated infection in Ethiopia: a systematic review and meta-analysis
**Short Title:**	Healthcare-associated infection
**Corresponding Author:**	Abebaw Alemu Yeshambel, MSc<br>Debre Tabor University<br>Debre Tabor, ETHIOPIA
**Keywords:**	Healthcare; Infection; Meta-analysis; Ethiopia
**Abstract:**	Background<br><br>Healthcare-related infection is a global threat because it leads to the emerging of multiple drug resistance microbial infections. Survey or surveillance data is required before or after the patient being discharged from health institutions. Different primary studies in Ethiopia showed that the magnitude of healthcare-associated infection was varying in different area at the same and/or similar study period. Hence, this systematic review and meta-analysis estimated the national prevalence and risk factors of healthcare-associated infection in Ethiopia.<br><br>Methods<br><br>We searched PubMed, Science Direct, Google Scholar and Addis Ababa university repository. The quality status of studies was appraised by using Joanna Brigg's Institute quality assessment scale. We employed funnel plot and Egger's regression test to assess publication bias. The national prevalence of healthcare-associated infection was estimated using a weight-inverse random-effect model meta-analysis. Finally, we did subgroup analysis to resolve the cause of statistical heterogeneity.<br><br>Results<br><br>A total of 18 studies with 14,240 patients were participated for prevalence estimation. The pooled prevalence of healthcare-associated infection was 16.96% (95% CI: 14.10-19.82). Those patients who had surgical procedure (AOR=3.61; 95% CI: 2.71–4.50) and diagnosis of underlying non-communicable disease (AOR=2.59; 95% CI: 1.63–3.55) were statistically significant risk factors of healthcare-associated infection.<br><br>Conclusions<br><br>The national prevalence of healthcare-associated infection remains high in Ethiopia. This review may help policy makers and program officers to design healthcare-associated infection preventive strategies. Infection prevention and control practice should be strengthened for patients who had surgical procedure and underlying non-communicable disease.
**Order of Authors:**	Abebaw Yeshambel, MSc
	Aklilu Endalamaw
	Wubet Alebachew
	Demeke Mesfin
	Demewoz Kefale
	Biniam Minuye

Powered by Editorial Manager® and ProduXion Manager® from Aries Systems Corporation

Additional Information:	
**Question**	**Response**
**Financial Disclosure**<br><br>Enter a financial disclosure statement that describes the sources of funding for the work included in this submission. Review the submission guidelines for detailed requirements. View published research articles from *PLOS ONE* for specific examples.<br><br>This statement is required for submission and **will appear in the published article** if the submission is accepted. Please make sure it is accurate.<br><br>**Unfunded studies**<br>Enter: *The author(s) received no specific funding for this work.*<br><br>**Funded studies**<br>Enter a statement with the following details:<br>• Initials of the authors who received each award<br>• Grant numbers awarded to each author<br>• The full name of each funder<br>• URL of each funder website<br>• Did the sponsors or funders play any role in the study design, data collection and analysis, decision to publish, or preparation of the manuscript?<br>• **NO** - Include this sentence at the end of your statement: *The funders had no role in study design, data collection and analysis, decision to publish, or preparation of the manuscript.*<br>• **YES** - Specify the role(s) played.<br><br>* typeset	The author(s) received no specific funding for this work.
**Competing Interests**<br><br>Use the instructions below to enter a competing interest statement for this submission. On behalf of all authors, disclose any competing interests that could be perceived to bias this work—acknowledging all financial support and any other relevant financial or non-financial competing interests.	The authors have declared that no competing interests exist.

Powered by Editorial Manager® and ProduXion Manager® from Aries Systems Corporation

This statement **will appear in the published article** if the submission is accepted. Please make sure it is accurate. View published research articles from *PLOS ONE* for specific examples.<br><br>**NO authors have competing interests**<br><br>Enter: *The authors have declared that no competing interests exist.*<br><br>**Authors with competing interests**<br><br>Enter competing interest details beginning with this statement:<br><br>*I have read the journal's policy and the authors of this manuscript have the following competing interests: [insert competing interests here]*<br><br>* typeset	
**Ethics Statement**<br><br>Enter an ethics statement for this submission. This statement is required if the study involved:<br><br>• Human participants<br>• Human specimens or tissue<br>• Vertebrate animals or cephalopods<br>• Vertebrate embryos or tissues<br>• Field research<br><br>Write "N/A" if the submission does not require an ethics statement.<br><br>General guidance is provided below. Consult the submission guidelines for detailed instructions. **Make sure that all information entered here is included in the Methods section of the manuscript.**	Not applicable

**Format for specific study types**<br><br>**Human Subject Research (involving human participants and/or tissue)**<br>• Give the name of the institutional review board or ethics committee that approved the study<br>• Include the approval number and/or a statement indicating approval of this research<br>• Indicate the form of consent obtained (written/oral) or the reason that consent was not obtained (e.g. the data were analyzed anonymously)<br><br>**Animal Research (involving vertebrate animals, embryos or tissues)**<br>• Provide the name of the Institutional Animal Care and Use Committee (IACUC) or other relevant ethics board that reviewed the study protocol, and indicate whether they approved this research or granted a formal waiver of ethical approval<br>• Include an approval number if one was obtained<br>• If the study involved *non-human primates*, add *additional details* about animal welfare and steps taken to ameliorate suffering<br>• If anesthesia, euthanasia, or any kind of animal sacrifice is part of the study, include briefly which substances and/or methods were applied<br><br>**Field Research**<br><br>Include the following details if this study involves the collection of plant, animal, or other materials from a natural setting:<br>• Field permit number<br>• Name of the institution or relevant body that granted permission	
**Data Availability**<br><br>Authors are required to make all data underlying the findings described fully available, without restriction, and from the time of publication. PLOS allows rare exceptions to address legal and ethical concerns. See the PLOS Data Policy and FAQ for detailed information.	Yes - all data are fully available without restriction

A Data Availability Statement describing where the data can be found is required at submission. Your answers to this question constitute the Data Availability Statement and **will be published in the article**, if accepted.<br><br>**Important:** Stating 'data available on request from the author' is not sufficient. If your data are only available upon request, select 'No' for the first question and explain your exceptional situation in the text box.<br><br>Do the authors confirm that all data underlying the findings described in their manuscript are fully available without restriction?	
**Describe where the data may be found in full sentences. If you are copying our sample text, replace any instances of XXX with the appropriate details.**<br><br>• If the data are **held or will be held in a public repository**, include URLs, accession numbers or DOIs. If this information will only be available after acceptance, indicate this by ticking the box below. For example: *All XXX files are available from the XXX database (accession number(s) XXX, XXX.).*<br>• If the data are all contained **within the manuscript and/or Supporting Information files**, enter the following: *All relevant data are within the manuscript and its Supporting Information files.*<br>• If neither of these applies but you are able to provide **details of access elsewhere**, with or without limitations, please do so. For example:<br><br>*Data cannot be shared publicly because of [XXX]. Data are available from the XXX Institutional Data Access / Ethics Committee (contact via XXX) for researchers who meet the criteria for access to confidential data.*<br><br>*The data underlying the results presented in the study are available from (include the name of the third party*	All relevant data are within the manuscript and its Supporting Information files

and contact information or URL).<br>• This text is appropriate if the data are owned by a third party and authors do not have permission to share the data.<br><br>* typeset	
Additional data availability information:	

Main mManuscript Click here to download Manuscript Manuscript PLOSE.docx

Prevalence and risk factors of healthcare-associated infection in Ethiopia: a systematic review and meta-analysis

Abebaw Yeshambel[1*], Aklilu Endalamaw[2], Wubet Alebachew[1], Demeke Mesfin[1], Demewoz Kefale[1], Biniam Minuye[1]

[1] Department of Paediatrics and Child Health Nursing, College of Health Sciences, Debre Tabor University, Debre Tabor, Ethiopia

[2] Department of Paediatrics and Child Health Nursing, School of Health Sciences, College of Medicine and Health Sciences, Bahir Dar University, Bahir Dar, Ethiopia

[*]**Corresponding authors e-mail:-** yeshambelabebaw@gmail.com (A Y); **PO.Box**-272

Abstract

Background: Healthcare-related infection is a global threat because it leads to the emerging of multiple drug resistance microbial infections. Survey or surveillance data is required before or after the patient being discharged from health institutions. Different primary studies in Ethiopia showed that the magnitude of healthcare-associated infection was varying in different area at the same and/or similar study period. Hence, this systematic review and meta-analysis estimated the national prevalence and risk factors of healthcare-associated infection in Ethiopia.

Methods: We searched PubMed, Science Direct, Google Scholar and Addis Ababa university repository. The quality status of studies was appraised by using Joanna Brigg's Institute quality assessment scale. We employed funnel plot and Egger's regression test to assess publication bias. The national prevalence of healthcare-associated infection was estimated using a weight-inverse random-effect model meta-analysis. Finally, we did subgroup analysis to resolve the cause of statistical heterogeneity.

Results: A total of 18 studies with 14,240 patients were participated for prevalence estimation. The pooled prevalence of healthcare-associated infection was 16.96% (95% CI: 14.10-19.82). Those patients who had surgical procedure (AOR=3.61; 95% CI: 2.71–4.50) and diagnosis of underlying non-communicable disease (AOR=2.59; 95% CI: 1.63–3.55) were statistically significant risk factors of healthcare-associated infection.

Conclusions: The national prevalence of healthcare-associated infection remains high in Ethiopia. This review may help policy makers and program officers to design healthcare-associated infection preventive strategies. Infection prevention and control practice should be strengthened for patients who had surgical procedure and underlying non-communicable disease.

Keywords: Healthcare; Infection; Meta-analysis; Ethiopia

Background

Healthcare-associated infection (HCAI) is preventable infection that a patient can encounter in healthcare facility while receiving medical care [1, 2]. It occurs after 48 hours of hospital admission, up to 3 days after discharge, or up to 30 days after operation when someone was admitted for reasons other than infection [2].

According to World Health Organization (WHO) 2019 HCAI fact sheet report, hundred million patients were affected each year globally. This report added that point prevalence of HCAI ranges 3.5-12% and 5.7-19.1% in developed and low-and middle-income countries, respectively [3]. The burden of HCAI was also reported in Sub-Saharan Africa [4] in general and specifically, in Botswana (13.4%) [5], South African (8%) [6], and Ethiopia (13 to 22%) [7-9].

Infection acquired in healthcare settings cause prolonged hospital stay, long term disability, increased microbial resistance to antibiotics, additional financial burden for health systems, high healthcare cost for patients and their families, and substantial mortality [10]. Hence, "*clean care the safer care*" program has been launched since 2004 with WHO patient safety directive, which was aimed to reduce HCAI through improving hand hygiene practice at the center of achieving

its aim [11]. Prevention guideline and infection prevention and control policy recommendations that was initiated by WHO have been implemented in Ethiopia.

Despite these efforts, studies done at different settings of the globe revealed that admission to surgical ward and hospital type [7], chest tube placement, prolonged hospital stays, patient on mechanical ventilation, previous hospitalization [8], pediatric patients, malnutrition and length of stay in hospital >5days [9] were contributing factors of HCAI.

Varieties of studies were conducted to determine the prevalence of HCAI in Ethiopia, but it showed great variation across geographical setting and variant time periods. Scattering on this, there was a need for nationally representative data on HCAI in the country. Moreover, the effect size of risk factors of HCAI weren't reported nationwide. Consequently, this systematic review and meta-analysis was aimed to estimate the national pooled prevalence and effect size of risk factors of HCAI in ~~Ethiopian~~.

Methods and materials

Reporting

The results of this review were reported based on the Preferred Reporting Items for Systematic Review and Meta-analysis statement (PRISMA) guideline [12] (Supporting file 1 PRISMA-P check list).

Inclusion and exclusion criteria

Cross-sectional, case-control and cohort studies were included. Those studies had reported the prevalence and/or at least one associated factor of HCAI. Studies published in English language were considered. There was no restriction of the study period. Citations without abstract and/or full-text, anonymous reports, editorials, and qualitative studies were excluded from the analysis.

Search strategy and information source

PubMed, Science Direct, Google Scholar and Addis Ababa University repository were searched. The core search terms and phrases were "prevalence", "incidence", "epidemiology",

"proportion", "magnitude", "burden", "associated factors", "risk factors", "predictors", "determinants", "healthcare-associated infections", "healthcare-acquired infections", and "nosocomial infections", "hospital acquired infections" and "Ethiopia. The search strategies were developed using different Boolean operators. Notably, to fit advanced PubMed database, the following search strategy was applied on February 5/2020: [(prevalence) OR incidence[MeSH Terms]) OR epidemiology[MeSH Terms]) OR proportion[MeSH Terms]) OR magnitude[MeSH Terms]) OR burden[MeSH Terms]) AND associated factors) OR risk factors[MeSH Terms]) OR predictors[MeSH Terms]) OR determinants[MeSH Terms]) AND healthcare-associated infections) OR healthcare-acquired infections[MeSH Terms]) OR nosocomial infections[MeSH Terms]) OR hospital acquired infections[MeSH Terms]) AND (Ethiopia)].

Study selection

Retrieved studies were exported to Endnote version 8 (Thomson Reuters, London) reference manager software to remove duplicate studies. Two independent reviewers screened the title and abstract. Disagreement was handled based on established article selection criteria. Two independent authors conducted the abstract and full-text review.

Quality assessment

Two independent authors appraised the quality of studies. The Joanna Briggs Institute (JBI) quality appraisal checklist was used [13]. Disagreement was resolved by interference of a third reviewer. The following items were used to appraise cohort studies: (i) similarity of groups, (ii) similarity of exposure measurement, (iii) validity and reliability of measurement, (iv) identification of confounder, (v) strategies to deal with confounder, (vi) appropriateness of groups/participants at the start of the study, (vii) validity and reliability of outcome measured, (viii) sufficiency of follow-up time, (ix) completeness of follow-up or descriptions of reason to loss to follow-up, (x) strategies to address incomplete follow-up, and (xi) appropriateness of statistical analysis. The items used to appraise case-control studies were: (i) comparable groups, (ii) appropriateness of cases and controls, (iii) criteria to identify cases and controls, (iv) standard measurement of exposure, (v) similarity in measurement of exposure for cases and controls, (vi) handling of confounders, (vii) strategies to handle confounder, (viii) standard assessment of outcome, (ix) appropriateness of duration for exposure, and (x) appropriateness of statistical

analysis. Cross-sectional studies were appraised based on: (i) inclusion criteria, (ii) description of study subject and setting, (iii) valid and reliable measurement of exposure, (iv) objective and standard criteria used, (v) identification of confounder, (vi) strategies to handle confounder, (vii) outcome measurement, and (viii) appropriate statistical analysis. All studies which got 50% and above on the quality scale were considered low risk.

Data extraction

Two independent reviewers extracted data using a structured data extraction form. Whenever variations of extracted data observed, the phase was repeated. If discrepancies between data extractors continued, third reviewer was involved. The name of the first author and year, study region, study design, target population, diagnostic methods, sample size, prevalence of HCAI, and adjusted odds ratio (AOR) of associated factors were collected.

Outcome measurement

HCAI was considered, when reported as infection(s) acquired while receiving medical care based on culture confirmation [9, 14-16], or clinical and laboratory methods [7, 17-30].

Statistical analysis

Publication bias was visually checked by funnel plot and objectively using Egger's regression test [31]. Heterogeneity of studies was quantified using the I-squared statistic, in which 25, 50, and 75% represented low, moderate and high heterogeneity respectively [32]. Pooled analysis was conducted using a weighted inverse variance random-effects model [33]. Subgroup analysis was done by region, study design, diagnostic method and sample size. Sensitivity analysis was employed to see the effect of single study on the overall estimation. Besides, the time-trend analysis was conducted to check whether variation through time is observed. STATA version 11 statistical software was used for meta-analysis.

Ethics approval and consent to participate

Not applicable because no primary data were collected.

Results

Literature search

The search strategy retrieved 611 articles from PubMed, 133 from Science Direct, 19 from Google Scholar and 3 from Addis Ababa university repository. After duplication removed, 680 remained. Sixty studies were screened for full-text review. Finally, 19 studies were used for meta-analysis (Figure 1).

The characteristics of included studies

Six studies were found in Addis Ababa [17-19, 21, 23, 30], five studies in Amhara region [7, 14, 24, 25, 27], five studies in Oromia [15, 16, 26, 28, 29], one study both in Addis Ababa and Southern Nation Nationalities and People Region (SNNPR) [20], one each in Tigray [22] and SNNPR [9]. Nine studies were done across all age group. Eight studies were done on adult population and one study was done on pediatric patients. Fourteen studies used clinical and laboratory methods to diagnosis HCAI while the remaining were culture confirmed. Four studies were conducted by using cohort study design, fourteen were cross sectional and only one was case-control study. Only six studies included > 1000 sample size (Table 1).

Table 1: Characteristics and quality status of the studies

First author year	Study region	Study design	Sample size	Prevalence	Quality status
Gedebu M. et al/1987 [17]	Addis Ababa	Cross sectional	2506	13.40	Low risk
Gedebu M. et al/1988 [18]	Addis Ababa	Cross sectional	700	17.00	Low risk
Habte-Gaber E. et al/1988 [19]	Addis Ababa	Cohort	1006	16.40	Low risk
Berhe N. et al/2001 [20]	Addis Ababa and SNNPR	Cohort	247	5.90	Low risk

Endalfer N. et al/2008 [21]	Addis Ababa	Cross sectional	854	9.00	Low risk
Tesfahun Z. et al/2009 [22]	Tigray region	Cross sectional	246	27.60	Low risk
Endalfer N. et al/2011 [23]	Addis Ababa	Cross sectional	215	35.80	Low risk
Melaku S. et al/2012 [24]	Amhara region	Cross sectional	1383	17.80	Low risk
Melaku S. eta al/2012 [25]	Amhara region	Cross sectional	1254	9.40	Low risk
Mulu W. et al/2013 [14]	Amhara region	Cross sectional	294	10.90	Low risk
Sahile T. eta al/2016 [26]	Oromia region	Cross sectional	500	35	Low risk
Yallew WW.et al/2016 [7]	Amhara region	Cross sectional	908	14.90	Low risk
Tolera M.et al/2018 [16]	Oromia region	Cross sectional	394	6.90	Low risk
Gashaw M. et al/2018 [15]	Oromia region	Cross sectional	1015	11.60	Low risk
Ali S. et al/2018 [29]	Oromia region	Cohort	1069	19.40	Low risk
Alemayehu T. et al/2019 [9]	SNNPR	Cross sectional	939	21.40	Low risk
Gebremeskel S. et al/2018 [30]	Addis Ababa	Cross sectional	410	19.80	Low risk
Yallew WW. et al/2017 [27]	Amhara region	Case-control	545		Low risk
Zewdu et al/2017 [28]	Oromia region	Cohort	300	14.00	Low risk

Note: SNNPR: Southern Nations Nationalities and Peoples Region

Quality of studies

The JBI quality appraisal criteria established for cross-sectional, case-control and cohort studies were used. The studies included in this systematic review and meta-analysis had no considerable risk. Therefore, all the studies were considered [7, 9, 14-30] (Table 1).

Meta-analysis

Publication bias

A funnel plot showed symmetrical distribution (Figure.2). Egger's regression test p-value was 0.328, which indicated the absence of publication bias.

Pooled prevalence of healthcare-associated infection

A total of ~~18~~ studies ~~with 14,240 patients~~ were participated for prevalence estimation. The estimated overall prevalence of HCAI is presented in a forest plot (Figure 3). The overall prevalence of HCAI was 16.96% (95% confidence interval (CI): 14.10-19.82).

Subgroup analysis

The subgroup analysis based on the region, study design, diagnostic method and sample size was done. Based on this, the prevalence of HCAI was found to be 27.6% in Tigray region, 18.2% diagnosed by both clinical and laboratory method, 17.83 % in the cross-sectional studies, 18.15% in studies using < 1000 study samples (Table 2).

Table 2: The pooled prevalence of HCAI, 95% CI and heterogeneity estimate with a p-value for the subgroup analysis

Variables	Characteristics	Pooled prevalence (95% CI)	I^2 (p-value)
**By region**	Addis Ababa	18.44% (14.02-22.86)	99% (0.000)
	Oromia	17.37% (9.2-25.56)	99.5% (0.000)
	Amhara	13.27% (9.00-17.52)	98.5% (0.000)

	Tigray	27.6% (25.37-29.83)	
	SNNPR	21.4% (20.3-22.5)	
	Addis Ababa & SNNPR	5.9% (4.25-7.55)	
**Study design**	Cross sectional	17.83% (14.39-21.27)	99.3% (0.000)
	Cohort	13.96% (8.78-19.14)	98.4% (0.000)
**Diagnostic method**	Clinical and laboratory	18.2% (14.85-21.51)	99.2% (0.000)
	Culture confirmed	12.71% (6.4-19.02)	99% (0.000)
**Sample size**	<1000	18.15% (13.28-23.03)	99.3% (0.000)
	≥1000	14.66% (11.72-17.59)	98.6% (0.000)

Note: SNNPR: Southern Nations Nationalities and Peoples Region

Sensitivity analysis

Endalafer N et al [23] and Sahile et al [26] had shown an impact on the overall estimation of the meta-analysis result (Figure 4).

Time trend analysis

The time-trend analysis showed that the prevalence of HCAI is increased from 13.4% in 1983 to 19.8% in 2017. However, the pooled prevalence from year to year is not increasing significantly (p-value = 0.620) (Figure 5).

Associated factors

Based on this review, HCAI in Ethiopian context is associated with socio-demographic-related, patient and healthcare-related factors.

Socio-demographic-related factors

Male sex (AOR=2.06; 95% CI: 1.01-4.22) as compared to female and age < 1 year (AOR=8.53; 95% CI:2.67-27.3) as compared to age > 56 years [30], patient age ≥ 51 years (AOR=6.38; 95% CI:1.15-35.14) as compared to age 11-20 years [14], age between 1-14 years (AOR=0.25; 95%

CI: 0.09-0.71) [27] and age between 18-30 years (AOR=0.54; 95% CI: 0.32-0.93) as compared to age <18 years [29], were more likely to had HCAI.

Patient and healthcare-related factors

Patients taking prophylaxis (AOR=1.76; 95% CI: 1.32-2.43) as compared to not taking prophylaxis [25], catheterized patients (AOR=18.9; 95% CI: 12.2-29.37) as compared to non-catheterized patients, duration of operation 90-150 minutes (AOR=11; 95% CI: 1.98 -60.83) as compared to < 30 minutes on operation [14], admission to surgical ward (AOR=2.86; 95% CI: 1.72-4.78) and admission to Felege Hiowt referral hospital Ethiopia (AOR=1.99; 95% CI: 1.36–2.93) as compared to admission to Gondar hospital Ethiopia [7] were at higher risk of developing HCAI. Patients who had central vascular catheter (AOR=6.92; 95% CI: 1.28–37.47) as compared to not having vascular catheter, taking antimicrobial (AOR=8.63; 95% CI: 3.11–23.95) as compared to not taking antimicrobial and presence of medical waste container in rooms (AOR=0.18; 95% CI: 0.03–0.97) as compared to no medical waste container in room [27] were more likely to develop HCAI. Hospital stay < 5 days (AOR=0.03; 95% CI:0.01–0.1 as compared to stayed >21 days was associated with HCAI [30]. Patients who were on mechanical ventilator (AOR=1.99; 95% CI: 1.06–3.74) as compared to patients who were not on mechanical ventilator [29] showed increased risk of HCAI.

The pooled effect of four studies [14, 23, 27, 29] showed that surgical procedure (AOR=3.61; 95% CI: 2.71–4.50) was found positively associated factor, but previous hospitalization [29, 30] and hospital stay > 5 days [9, 14] had insignificant association with HCAI. The pooled effect of four studies [14, 25, 27, 29] showed that patients who had underlying disease (AOR=2.59; 95% CI: 1.63–3.55) as compared to patients with no underlying disease were more likely to had HCAI (Figure 6).

Discussion

The Sustainable Development Goal (SDG) goal-3 is implementing to ensure access to quality essential healthcare service in every country [34]. However, HCAI is a major challenge of the SDG of infection prevention and control in health systems, which fail to build strong principles

to reduce the risks and spread of nosocomial infection [35]. HCAI is among preventable cause of morbidity, mortality and spending health system cost.

This meta-analysis estimated the national prevalence of HCAI and contextual associated factors in Ethiopia. Accordingly, the national pooled prevalence of HCAI was 16.96% (95% CI: 14.10-19.82). This result was higher than studies done in China (3.12%) [36], Morocco (10.3%) [37], Botswana (13.54%) [5] and south Africa (7.67%) [6]. The possible reasons for high prevalence in this study might be very low hand hygiene practice by physicians, low adherence to infection prevention practice, low level of job satisfaction, morally distressed nurses, resources constraints, low implementation of nursing process and less attention given to HCAI.

Perform hand hygiene before patient contact is vital to prevent HCAI. However, only 7% of physicians working at two university hospitals in Addis Ababa Ethiopia performed hand hygiene before patient contact [38], as a result acquisition of HCAI from these healthcare personnel might be high. Evidence also showed that 35% of nurses were non-adherent to infection prevention practice in southwest Ethiopia [39], high HCAI could be explained by this low adherence to infection prevention. Besides, nearly 68% of health professionals were less satisfied of their work in Amhara region [40], unsatisfied health professionals are less likely to deliver quality healthcare, so nosocomial infection was inevitably high. Additionally, about 84% of nurses were morally distressed in northwestern part of the country [41], as morally less prepared nurses were unable to deliver quality nursing care, consequently HCAI was found high.

Resource constraints could increase HCAI in the country, because lack of hand hygiene agents and sinks were reported as hindering factors of infection prevention practice in Addis Ababa Ethiopia [38]. Implementation of nursing process was below half (49%) in northwest part of the country [42], so nursing intervention would not be planed for patients at risk of nosocomial infection, hence HCAI was found higher. Furthermore, healthcare providers, patients and/or families are more curious for primary reason of admission or healthcare visit, so less attention is given for HCAI which could justify high prevalence of HCAI in the country.

Moreover, the time trend analysis showed that HCAI was slightly increasing in Ethiopia. The possible reasons might be more emphasis given for healthcare coverage than quality, increase in

technological advancement and over utilization of invasive procedures. Evidence revealed that advance in life saving medical practices increase exposure to invasive procedures, which increase the occurrences of nosocomial infections [43]. On top of this, nurse's burnout might contribute for increasing trend. Evidence in United states (US) revealed that nurse's burnout was found as a single most important associated factor for increased nosocomial urinary tract infection (UTI) and surgical site infection (SSI) [44].

The current meta-analysis revealed that patients who had surgical procedure and underlying non-communicable disease were more likely to acquire HCAI.

Patients who had surgical procedure were associated with nearly four times increased odds of HCAI in this study. The finding is in line with previous studies done in south Africa [6] and Poland [45]. The reason for observed association could be explained by less compliance to hand hygiene practice, and high prevalence (25.22%) of surgical site infection [46] in Ethiopia. Compliance to hand hygiene practice is pivotal for prevention and control of nosocomial infection, but only 3.6% keep hand hygiene before performing aseptic procedures at Debre Birhan referral hospital, central Ethiopia [47]. The overall compliance to hand hygiene practice was 18.7% [48] at Hiwot Fana specialized and 22% [47] at Debre Birhan referral hospitals of Ethiopia in 2017. Consequently, unable to keep and maintain hand hygiene practice increases acquisition of HCAI.

The odds of patients having underlying non-communicable disease were nearly three times more at risk to develop HCAI. This finding is supported by studies that reported HCAI was positively associated with diabetes mellitus [49] and underlying renal disease [50]. The possible explanations for observed association in the current study might be due to high prevalence of underlying diseases and immunosuppression effects of these diseases. A meta-analysis studies showed that high prevalence (6.5%) of diabetes mellitus in Ethiopia [51] and immunosuppression was identified as a risk factor for HCAI [49].

Strength and limitation

This systematic review and meta-analysis was the national estimation conducted in Ethiopia. It may lack national representativeness because no data were found from Benishangul Gumuz, Afar, Gambella, Somalia, Dire Dawa, and Harare regions of Ethiopia. Time-trend analysis might not reflect the exact trend because all the years didn't have reported data.

Conclusions

The prevalence of healthcare-associated infection in Ethiopia remains high. This review may help policy-makers and program officers to design interventions on preventing healthcare-associated infection. The existing infection prevention and control practice for patients who had surgical procedure and underlying non-communicable disease should also be strengthened.

Abbreviations

AOR: Adjusted Odds Ratio; CI: Confidence Interval; HCAI: Healthcare-Associated Infection; LMIC: Low and Middle Income Country; SNNPR: Sothern Nations Nationalities and Peoples Region; WHO: World Health Organization

Acknowledgments

Not applicable

References

1. What are healthcare-associated infections? [Internet]. [cited 01 Feb 2020]. Available from: https://apic.org/monthly_alerts/what-are-healthcare-associated-infections/.

2. Infections caught in the hospital [Internet]. [cited 02 Feb 2020]. Available from: https://www.healthline.com/health/hospital-acquired-nosocomial-infections.

3. Health care-associated infections FACT SHEET [Internet]. [cited 02 Feb 2020]. Available from: https://www.who.int/gpsc/country_work/gpsc_ccisc_fact_sheet_en.pdf.

4. Rothe C, Schlaich C, Thompson S. Healthcare-associated infections in sub-Saharan Africa. Journal of Hospital Infection. 2013;85(4):257-67.

5. Mpinda-Joseph P, Anand Paramadhas BD, Reyes G, Maruatona MB, Chise M, Monokwane-Thupiso BB, et al. Healthcare-associated infections including neonatal bloodstream infections in a leading tertiary hospital in Botswana. Hospital Practice. 2019;47(4):203-10.

6. Nair A, Steinberg W, Habib T, Saeed H, Raubenheimer J. Prevalence of healthcare-associated infection at a tertiary hospital in the Northern Cape Province, South Africa. South African Family Practice. 2018;60(5):162-7.

7. Yallew WW, Kumie A, Yehuala FM. Point prevalence of hospital-acquired infections in two teaching hospitals of Amhara region in Ethiopia. Drug, healthcare and patient safety. 2016;8:71.

8. Ali S, Birhane M, Bekele S, Kibru G, Teshager L, Yilma Y, et al. Healthcare associated infection and its risk factors among patients admitted to a tertiary hospital in Ethiopia: longitudinal study. Antimicrobial Resistance & Infection Control. 2018;7(1):2.

9. Alemayehu T, Tadesse E, Ayalew S, Nigusse B, Yeshitila B, Amsalu A, et al. HIGH BURDEN OF NOSOCOMIAL INFECTIONS CAUSED BY MULTI-DRUG RE-SISTANT PATHOGENS IN PEDIATRIC PATIENTS AT HAWASSA UNIVERSITY COMPREHENSIVE SPECIALIZED HOSPITAL. 2019.

10. World Health Organization. Report on the Burden of Endemic Health Care-Associated Infection Worldwide. Geneva, Switzerland; 2011.

11. Allegranzi B, Storr J, Dziekan G, Leotsakos A, Donaldson L, Pittet D. The first global patient safety challenge “clean care is safer care”: from launch to current progress and achievements1. Journal of Hospital Infection. 2007;65:115-23.

12. Shamseer L, Moher D, Clarke M, Ghersi D, Liberati A, Petticrew M, et al. Preferred reporting items for systematic review and meta-analysis protocols (PRISMA-P) 2015: elaboration and explanation. Bmj. 2015;349.

13. Peters MD, Godfrey CM, McInerney P, Soares CB, Khalil H, Parker D. The Joanna Briggs Institute reviewers' manual 2015: methodology for JBI scoping reviews2015. Available from: https://www.who.int/nutrition/publications/globaltargets2025_policybrief_lbw/en/.

14. Mulu W, Kibru G, Beyene G, Damtie H. Associated risk factors for postoperative nosocomial infections among patients admitted at Felege Hiwot Referral Hospital, Bahir Dar, Northwest Ethiopia. Clinical medicine & research. 2013;2(6):140-7.

15. Gashaw M, Berhane M, Bekele S, Kibru G, Teshager L, Yilma Y, et al. Emergence of high drug resistant bacterial isolates from patients with health care associated infections at Jimma University medical center: a cross sectional study. Antimicrobial resistance and infection control. 2018;7:138.

16. Tolera M, Abate D, Dheresa M, Marami D. Bacterial nosocomial infections and antimicrobial susceptibility pattern among patients admitted at Hiwot Fana Specialized University Hospital, Eastern Ethiopia. Advances in medicine. 2018;2018.

17. Gedebou M, Kronvall G, Habte-Gabr E, Ringertz S. The bacteriology of nosocomial infections at Tikur Anbessa Teaching Hospital, Addis Ababa. Acta pathologica, microbiologica, et immunologica Scandinavica Section B, Microbiology. 1987;95(6):331-6.

18. Gedebou M, Habte-Gabr E, Kronvall G, Yoseph S. Hospital-acquired infections among obstetric and gynaecological patients at Tikur Anbessa Hospital, Addis Ababa. The Journal of hospital infection. 1988;11(1):50-9.

19. Habte-Gabr E, Gedebou M, Kronvall G. Hospital-acquired infections among surgical patients in Tikur Anbessa Hospital, Addis Ababa, Ethiopia. Am J Infect Control. 1988;16(1):7-13.

20. Berhe N, Hailu A, Abraham Y, Tadesse Y, Breivik K, Abebe Y. Inter-current and nosocomial infections among visceral leishmaniasis patients in Ethiopia: an observational study. Acta tropica. 2001;80(2):87-95.

21. Endalafer N. Bacterial Nosocomial Infections and Their Antimicrobial Susceptibility Patterns in Surgical Wards and Surgical Intensive Care Unit (SICU) Of Tikur Anbessa University Hospital Addis Ababa, Ethiopia: Addis Ababa University; 2008.

22. Tesfahunegn Z, Asrat D, Woldeamanuel Y, Estifanos K. Bacteriology of surgical site and catheter related urinary tract infections among patients admitted in Mekelle Hospital, Mekelle, Tigray, Ethiopia. Ethiop Med J. 2009;47(2):117-27.

23. Endalafer N, Gebre-Selassie S, Kotiso B. Nosocomial bacterial infections in a tertiary hospital in Ethiopia. Journal of Infection Prevention. 2011;12(1):38-43.

24. Melaku S, Gebre-Selassie S, Damtie M, Alamrew K. Hospital acquired infections among surgical, gynaecology and obstetrics patients in Felege-Hiwot referral hospital, Bahir Dar, northwest Ethiopia. Ethiop Med J. 2012;50(2):135-44.

25. Melaku S, Kibret M, Abera B, Gebre-Sellassie S. Antibiogram of nosocomial urinary tract infections in Felege Hiwot referral hospital, Ethiopia. Afr Health Sci. 2012;12(2):134-9.

26. Sahile T, Esseye S, Beyene G, Ali SJIJTDH. Post-surgical infection and antibiotic susceptibility patterns of bacteria isolated from admitted patients with signs of infection at Jimma University specialized hospital, Jimma, Ethiopia. 2016;17(4):1-12.

27. Yallew WW, Kumie A, Yehuala FM. Risk factors for hospital-acquired infections in teaching hospitals of Amhara regional state, Ethiopia: A matched-case control study. PLoS One. 2017;12(7):e0181145.

28. Zewdu. A. Health Care Associated Infection at Adama Hospital Medical College, Adama Oromia, Ethiopia. Addis Ababa: Addis Ababa University 2017.

29. Ali S, Birhane M, Bekele S, Kibru G, Teshager L, Yilma Y, et al. Healthcare associated infection and its risk factors among patients admitted to a tertiary hospital in Ethiopia: longitudinal study. Antimicrobial resistance and infection control. 2018;7:2.

30. Gebremeskel S. Management of Hospital-Acquired Infections among Hospitalized Patients at Zewditu Memorial Hospital, Addis Ababa, Ethiopia: A Prospective Study: Addis Ababa Universty; 2018.

31. Rothstein HR, Sutton AJ, Borenstein M. Publication bias in meta-analysis. 2005.

32. Ioannidis JP. Interpretation of tests of heterogeneity and bias in meta- analysis. Journal of evaluation in clinical practice. 2008;14(5):951-7.

33. Borenstein M, Hedges LV, Higgins JP, Rothstein HR. A basic introduction to fixed-effect and random- effects models for meta- analysis. Research synthesis methods. 2010;1(2):97-111.

34. World Health Organization. Health in 2015: from MDGs, millennium development goals to SDGs, sustainable development goals: World Health Organization; 2015.

35. Allegranzi B, Kilpatrick C, Storr J, Kelley E, Park BJ, Donaldson L. Global infection prevention and control priorities 2018–22: a call for action. The Lancet Global Health. 2017;5(12):e1178-e80.

36. Wang J, Liu F, Tartari E, Huang J, Harbarth S, Pittet D, et al. The prevalence of healthcare-associated infections in mainland China: a systematic review and meta-analysis. 2018;39(6):701-9.

37. Razine R, Azzouzi A, Barkat A, Khoudri I, Hassouni F, Chefchaouni AC, et al. Prevalence of hospital-acquired infections in the university medical center of Rabat, Morocco. International archives of medicine. 2012;5(1):26.

38. Tenna A, Stenehjem EA, Margoles L, Kacha E, Blumberg HM, Kempker RRJIC, et al. Infection control knowledge, attitudes, and practices among healthcare workers in Addis Ababa, Ethiopia. 2013;34(12):1289-96.

39. Bekele I, Yimam I, Akele GJIR. Adherence to Infection prevention and factors among nurses in jimma university medical center. 2018;14(2):1-7.

40. Temesgen K, Aycheh MW, Leshargie CTJH, outcomes qol. Job satisfaction and associated factors among health professionals working at Western Amhara Region, Ethiopia. 2018;16(1):65.

41. Berhie AY, Tezera ZB, Azagew AWJPR, Management B. Moral Distress and Its Associated Factors Among Nurses in Northwest Amhara Regional State Referral Hospitals, Northwest Ethiopia. 2020;13:161.

42. Semachew AJBrn. Implementation of nursing process in clinical settings: the case of three governmental hospitals in Ethiopia, 2017. 2018;11(1):173.

43. Bereket W, Hemalatha K, Getenet B, Wondwossen T, Solomon A, Zeynudin A, et al. Update on bacterial nosocomial infections. European review for medical and pharmacological sciences. 2012;16(8):1039-44.

44. Cimiotti JP, Aiken LH, Sloane DM, Wu ES. Nurse staffing, burnout, and health care–associated infection. American Journal of Infection Control. 2012;40(6):486-90.

45. Deptuła A, Trejnowska E, Ozorowski T, Hryniewicz WJJoHI. Risk factors for healthcare-associated infection in light of two years of experience with the ECDC point prevalence survey of healthcare-associated infection and antimicrobial use in Poland. 2015;90(4):310-5.

46. Birhanu Y, Endalamaw A. Surgical site infection and pathogens in Ethiopia: a systematic review and meta-analysis. Patient Safety in Surgery. 2020;14(1):1-8.

47. Kolola T, Gezahegn T. A twenty-four-hour observational study of hand hygiene compliance among health-care workers in Debre Berhan referral hospital, Ethiopia. Antimicrobial resistance and infection control. 2017;6(1):109.

48. Awoke N, Geda B, Arba A, Tekalign T, Paulos KNr, practice. Nurses Practice of Hand Hygiene in Hiwot Fana Specialized University Hospital, Harari Regional State, Eastern Ethiopia: Observational Study. Nursing research. 2018;2018.

49. Rodríguez-Acelas AL, de Abreu Almeida M, Engelman B, Cañon-Montañez W. Risk factors for health care–associated infection in hospitalized adults: Systematic review and meta-analysis. American Journal of Infection Control. 2017;45(12):e149-e56.

50. Greene MT, Chang R, Kuhn L, Rogers MA, Chenoweth CE, Shuman E, et al. Predictors of hospital-acquired urinary tract–related bloodstream infection. 2012;33(10):1001-7.

51. Bishu KG, Jenkins C, Yebyo HG, Atsbha M, Wubayehu T, Gebregziabher M. Diabetes in Ethiopia: A systematic review of prevalence, risk factors, complications, and cost. Obesity Medicine. 2019;15:100132.

List of figures

Figure 1: The study selection process

Figure 2: Funnel plot for publication bias, LN of proportion (X-axis) with its standard error of LN of proportion (Y-axis)

Figure 3: Forest plot of the pooled prevalence (ES) of HCAI with corresponding 95% CIs

Figure 4: The sensitivity analysis showed the pooled prevalence when the studies omitted step by step

Figure 5: Time-trend analysis of the prevalence of HCAI in Ethiopia from 1983 to 2017

Figure 6: The pooled effect of previous hospitalization, hospital stay, had surgical procedure and diagnosis of underlying non communicable diseases on HCAI

List of tables

Table 1: Characteristics and quality status of the studies

Table 2: The pooled prevalence of HCAI, 95% CI and heterogeneity estimate with a p-value for the subgroup analysis

Figure 1

Click here to download Figure Figure 1.tif

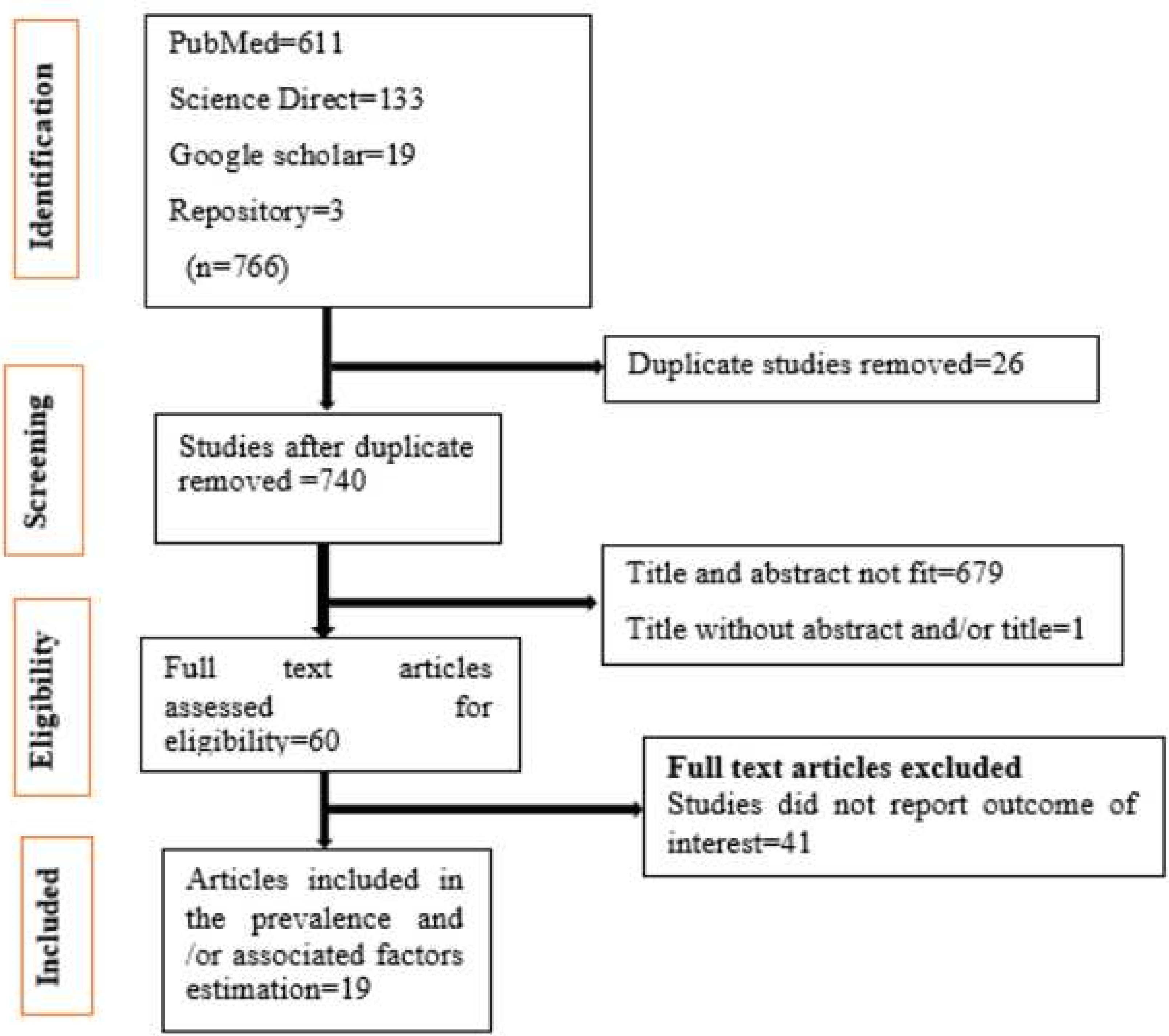

Figure 2

Click here to download Figure Figure 2.tif

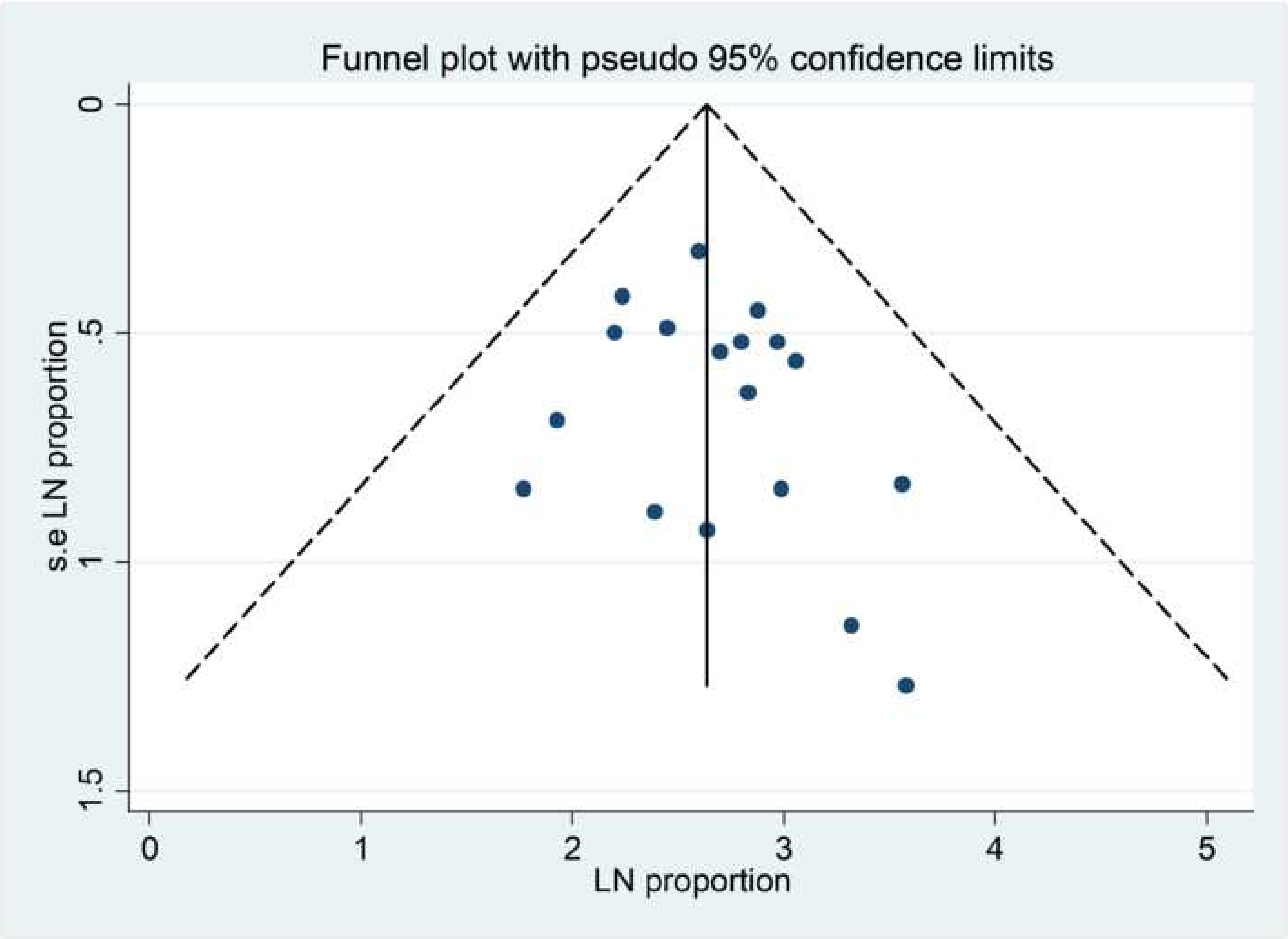

Figure 3

Click here to download Figure Figure 3.tif

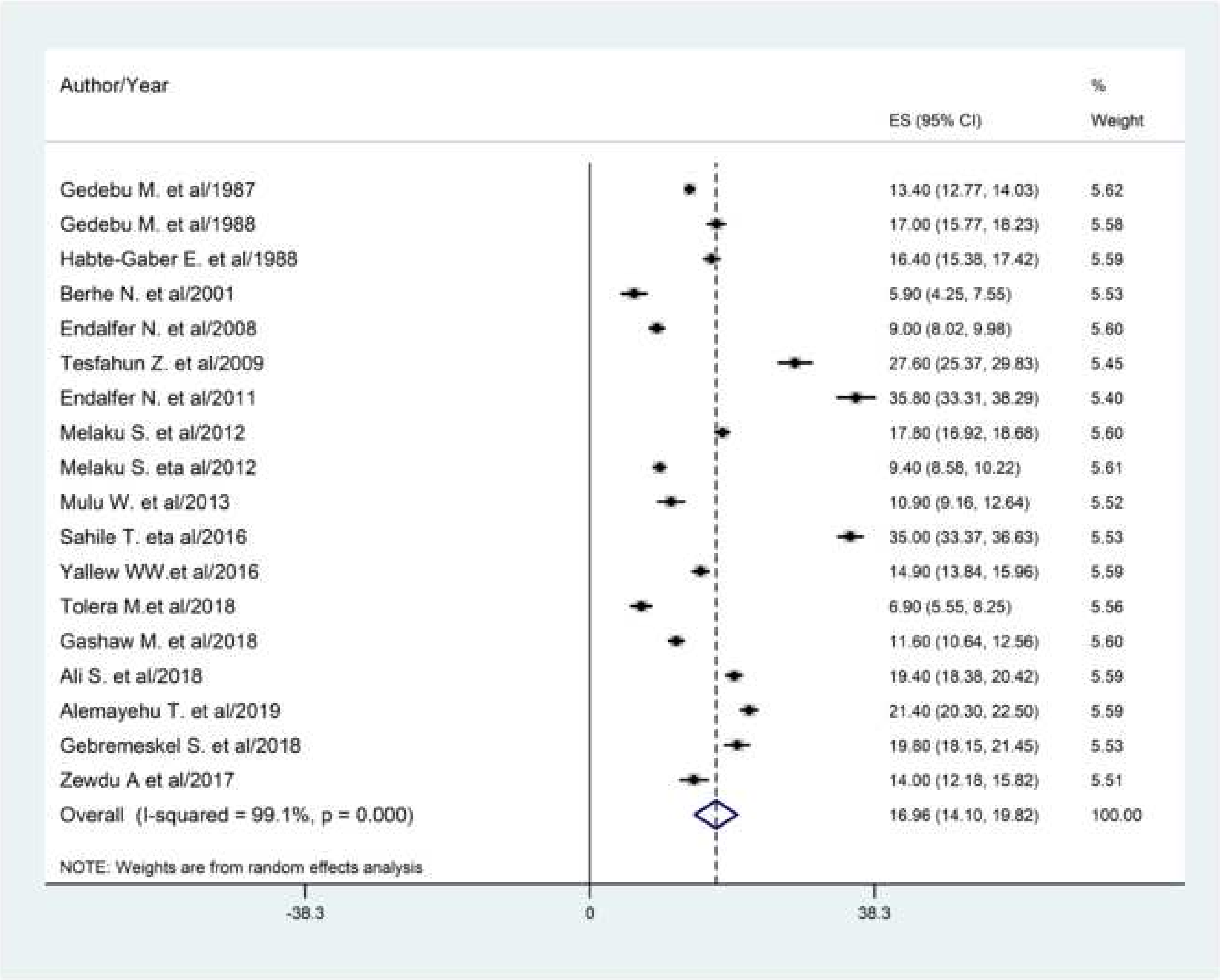

Figure 4

Click here to download Figure Figure 4.tif

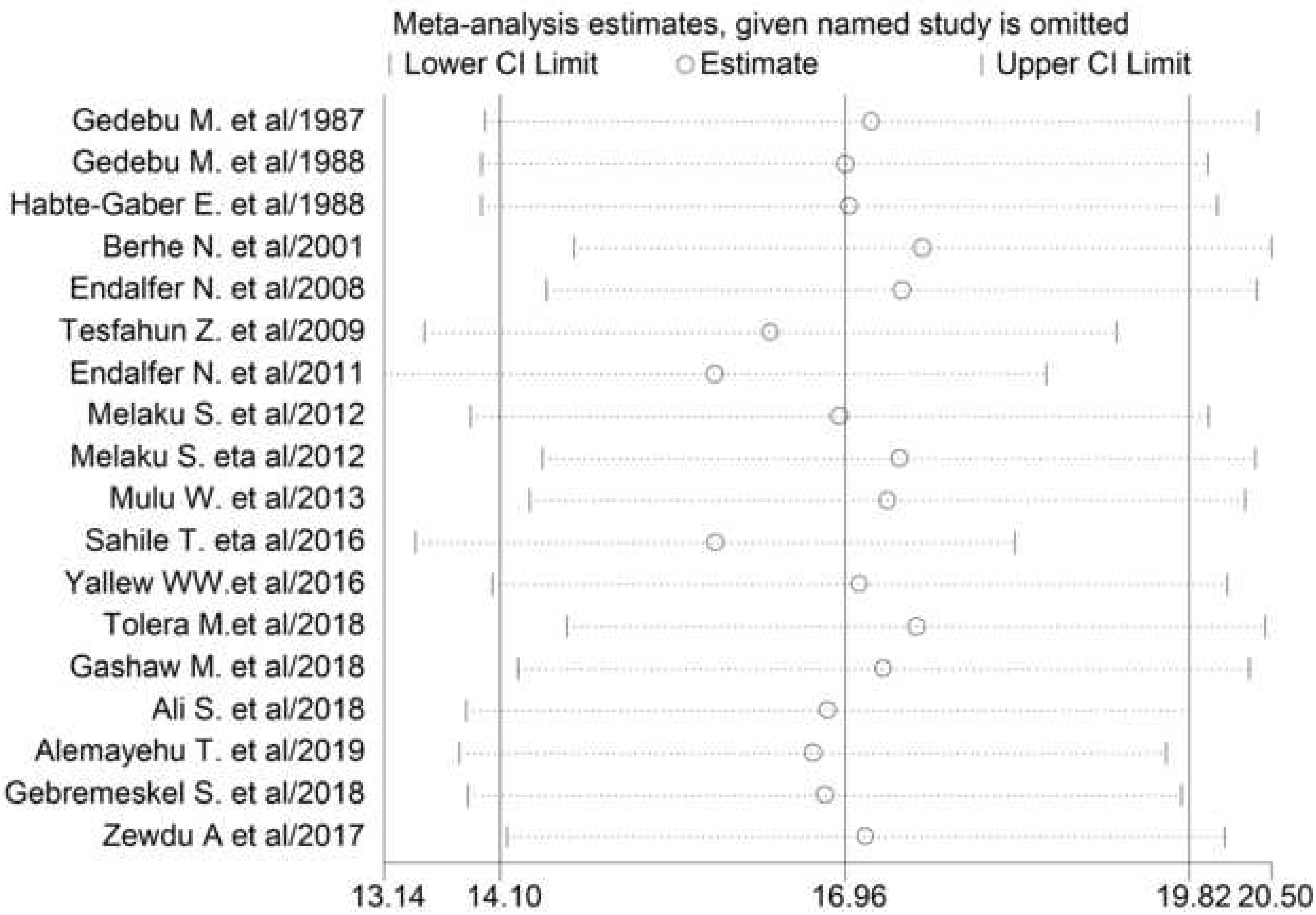

Figure 5

Click here to download Figure Figure 5.tif

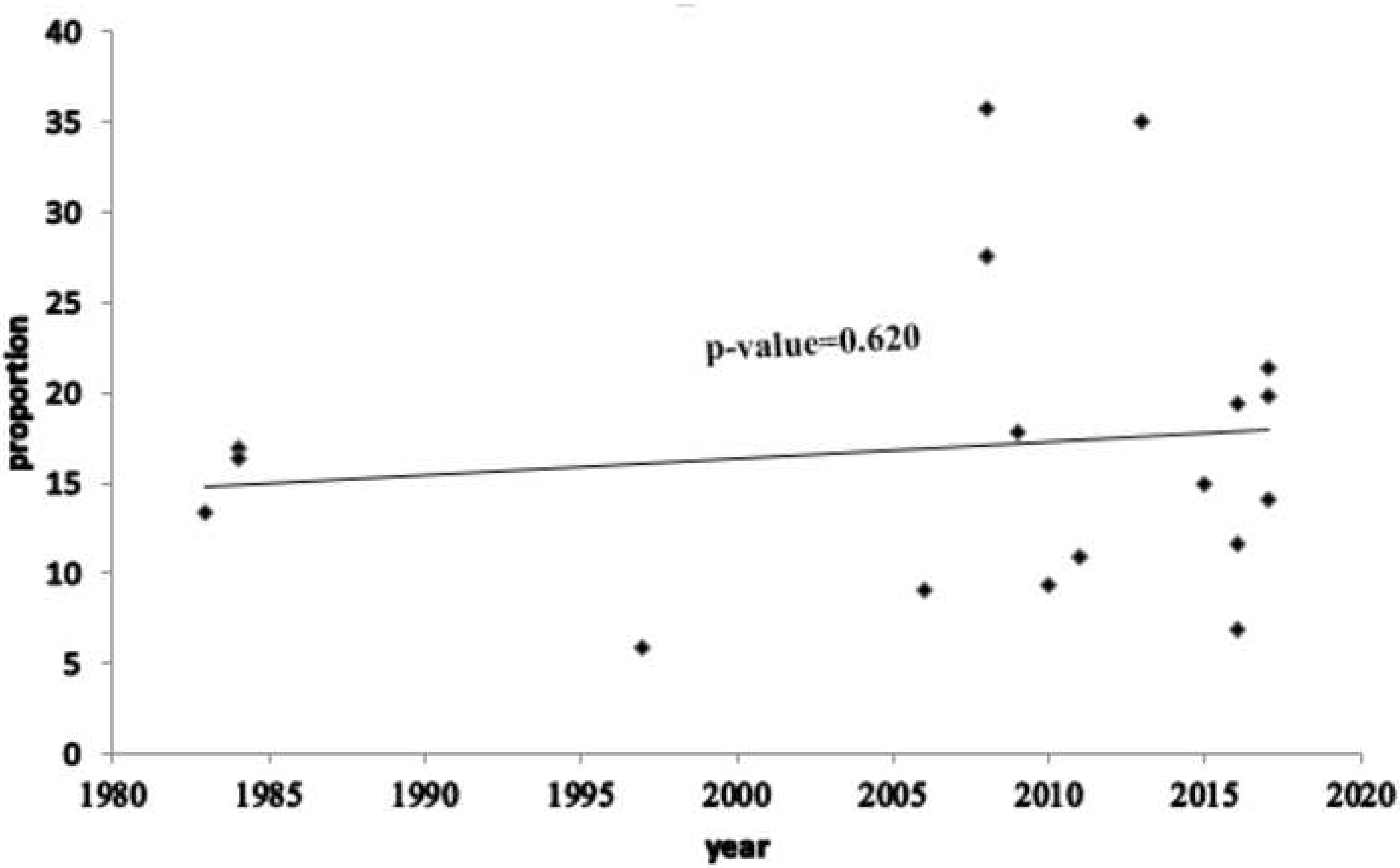

Figure 6

Click here to download Figure Figure 6.tif

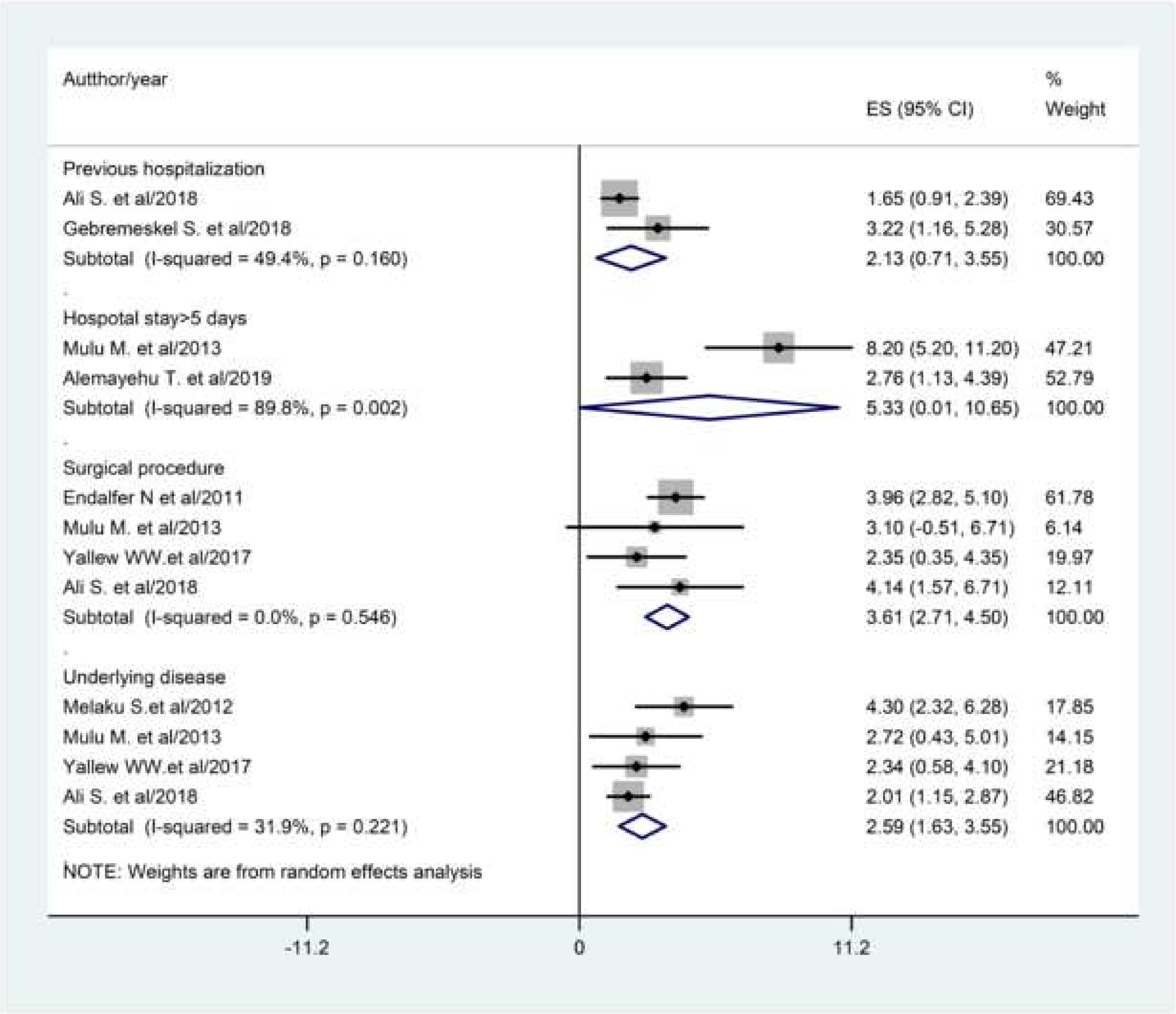

Supporting Information 1

Click here to access/download
Supporting Information
PRISMA-P-checklist.doc
